# Elevated N‐glycosylated cathepsin L impairs oocyte function and contributes to oocyte senescence during reproductive aging

**DOI:** 10.1111/acel.14397

**Published:** 2024-11-04

**Authors:** Kemei Zhang, Rui Xu, Lu Zheng, Hong Zhang, Zhang Qian, Chuwei Li, Mengqi Xue, Zhaowanyue He, Jinzhao Ma, Zhou Li, Li Chen, Rujun Ma, Bing Yao

**Affiliations:** ^1^ Department of Reproductive Medicine Jinling Clinical Medical College, Nanjing Medical University Nanjing China; ^2^ Department of Reproductive Medicine Jinling Hospital, Affiliated Hospital of Medical School, Nanjing University Nanjing China; ^3^ Department of Reproductive Medicine Jinling Hospital, School of Medicine, Jiangsu University Zhenjiang China

**Keywords:** cathepsin L, lysosome, meiosis, mitochondrial function, N‐glycosylation, oocyte senescence, reproductive aging

## Abstract

Age‐related declines in oocyte quality and ovarian function are pivotal contributors to female subfertility in clinical settings. Yet, the mechanisms driving ovarian aging and oocyte senescence remain inadequately understood. The present study evaluated the alterations in N‐glycoproteins associated with ovarian aging and noted a pronounced elevation in N221 glycopeptides of cathepsin L (Ctsl) in the ovaries of reproductive‐aged mice (8–9 months and 11–12 months) compared to younger counterparts (6–8 weeks). Subsequent analysis examined the involvement of Ctsl in oocyte aging and demonstrated a significant elevation in Ctsl levels in aged oocytes. Further, it was revealed that the overexpression of Ctsl in young oocytes substantially diminished their quality, while oocytes expressing an N221‐glycosylation mutant of Ctsl did not suffer similar quality degradation. This finding implies that the N221 glycosylation of Ctsl is pivotal in modulating its effect on oocyte health. The introduction of a Ctsl inhibitor into the culture medium restored oocyte quality in aged oocytes by enhancing mitochondrial function, reducing accumulated reactive oxygen species (ROS), lowering apoptosis, and recovering lysosome capacity. Furthermore, the targeted downregulation of Ctsl using siRNA microinjection in aged oocytes enhanced fertilization capability and blastocyst formation, affirming the role of Ctsl knockdown in fostering oocyte quality and embryonic developmental potential. In conclusion, these findings underscore the detrimental effects of high expression of N‐glycosylated Ctsl on oocyte quality and its contribution to oocyte senescence, highlighting it as a potential therapeutic target to delay ovarian aging and enhance oocyte viability.

## Abstract 2

Ctsl is a protein synthesized in the cytoplasm of oocytes, where it undergoes N‐glycosylation at the N221 site. In the process of oocyte aging, there is a notable accumulation of N‐glycosylated Ctsl, which adversely impacts oocyte quality by impairing mitochondrial function, increasing reactive oxygen species (ROS) production, promoting apoptosis, and compromising lysosomal capacity. These alterations ultimately have detrimental effects on meiotic maturation and embryonic development.

## Graphical abstract

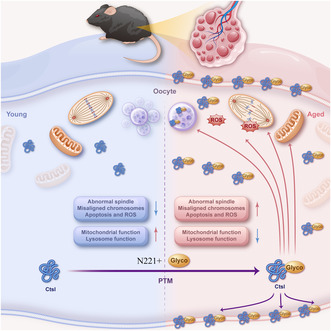

## 1 INTRODUCTION

Epidemiological studies indicate a rapid decline in female reproductive function beginning as early as 35 years of age, characterized by diminished ovarian reserve and impaired oocyte quality (Laisk et al., 2019). Age has been pinpointed as a critical, independent risk factor influencing ovarian function (Crawford & Steiner, 2015). Moreover, the societal shift towards postponing childbearing has increasingly made infertility a pressing global issue. Ovarian aging not only affects reproductive health but is also linked to several systemic conditions, such as osteoporosis, dyspareunia, and cardiovascular and cerebrovascular disorders. Thus, extensive research over recent decades has led to significant advancements in understanding ovarian failure and oocyte aging (M. Li et al., 2021; S. Wang et al., 2020). Multiple studies have identified several key factors contributing to these phenomena, including mitchondrial dysfunction (Smits et al., 2023), excessive accumulation of reactive oxygen species (ROS) (L. Wang et al., 2021), disrupted autophagy (Jin et al., 2022), inflammation, and fibrosis due to collagen fiber deposition (Umehara et al., 2022). Nonetheless, the precise mechanisms underlying the decline in ovarian function and oocyte quality associated with natural aging are still not fully elucidated.

Posttranslational modifications (PTMs) confer a crucial role in the regulation of gene expression, ensuring the proper structural integrity and function of proteins in the ovarian environment. These modifications include but are not limited to phosphorylation, dephosphorylation, methylation, acetylation, ubiquitination, SUMOylation, and de‐SUMOylation (Akera, 2023; Briley et al., 2023). Among these PTMs, glycosylation is particularly significant in the ovarian context, with N‐linked glycosylation (glycans attached to asparagine residues) and O‐linked glycosylation (glycans attached to serine or threonine residues) being the most prevalent (H. Li et al., 2020). N‐glycosylation is typically associated with proteins that are secreted or are components of membranes within the cytomembrane or organelles such as the lysosome, Golgi apparatus, and endoplasmic reticulum (Moremen et al., 2012; Ohtsubo & Marth, 2006). As a crucial endocrine organ, the ovary orchestrates hormone production and regulation via hormone receptors and growth factors, many of which are subject to glycosylation. It is imperative to recognize that appropriate N‐glycosylation is essential for the maturation of oocytes and overall female reproductive function. The anti‐Müllerian hormone (AMH), produced and secreted by granulosa cells, has been identified as a vital marker of ovarian performance (di Clemente et al., 2021). Furthermore, research has demonstrated that missense mutations in Dpagt1, the enzyme responsible for catalyzing the initial step of protein N‐glycosylation, can result in subfertility in female mice (H. Li et al., 2020). Nevertheless, our understanding of the systematic variations in N‐glycosylated proteins and the levels of N‐glycosylated modifications during ovarian failure and oocyte aging remains considerably limited.

In present research, we evaluated the alterations in N‐glycoproteins in the ovaries of three different age groups: 6–8 weeks (6–8 W), 8–9 months (8–9 M), and 11–12 months (11–12 M) via mass spectrometry (MS)‐based quantitative N‐glycoproteomics. Our findings suggested hyperglycosylation in the ovaries of mice aged 8–9 M and 11–12 M when compared to the 6–8 W mice. Further bioinformatics analysis of the differentially expressed (DE) N‐glycosylated proteins pinpointed significant enrichment in pathways related to lysosome, phagosome, and autophagy. Interestingly, among DE glycoproteins in the lysosome pathway, cathepsin L (Ctsl) emerged as significantly upregulated in the ovaries and oocytes of the 8–9 M and 11–12 M mice compared to those aged 6–8 W.

Ctsl, a cysteine protease belonging to the cathepsins family (Patel et al., 2018), has been implicated in a variety of biological events, including immune response, autophagy, cellular proliferation, and differentiation (Duncan et al., 2008). Several studies have reported the involvement of Ctsl in ovarian function. Robker et al. demonstrated that Ctsl could be induced in granulosa cells in a progesterone (Prog) receptor‐dependent manner and took part in follicular rupture (Robker et al., 2000). Besides, it was proved that Ctsl plays a pivotal role in regulating oocyte meiosis and early embryonic development (Ezz et al., 2024). Despite these roles, the specific functions of Ctsl in the ovary, particularly during ovarian failure and oocyte aging, remain inadequately understood.

This study initially verified the critical role of the N221 glycosylation site on Ctsl in influencing oocyte aging through targeted overexpression experiments. Then, the role of Ctsl in oocyte maturation was further explored utilizing Ctsl inhibitors and knockdown (KD) approaches. These investigative efforts shed light on the functional relevance and mechanistic underpinnings of Ctsl in ovarian function and oocyte quality, uncovering its detrimental impact on the meiotic maturation of oocytes and subsequent embryonic developmental competence. Importantly, our findings underscore the pivotal role of the N‐glycosylation of Ctsl in mediating these effects.

## 2 RESULTS

### 2.1 Decline in ovarian function and follicle numbers at different developmental stages in aging mice

Comparative analyses were initially performed on mouse body weight, ovary weight, and ovary index among three age groups (6–8 W, 8–9 M, and 11–12 M) (Figure S1A–D). Both body weights and ovary weights in 8–9 M and 11–12 M groups were greater than that of 6–8 W mice (Figure S1B,C). However, there were not significant differences in ovary indexes among three age groups (Figure S1D). Follicular assessments at different developmental stages were subsequently performed (Figure S1E), revealing a substantial reduction in the numbers of primordial, primary, secondary, preantral, and antral follicles in the 8–9 M and 11–12 M groups, especially in the 11–12 M group (Figure S1F–J). The results meant that there were microscopic structural changes in ovaries of 8–9 M and 11–12 M mice when comparing with 6–8 W mice, but not ovary index.

**FIGURE 1:**
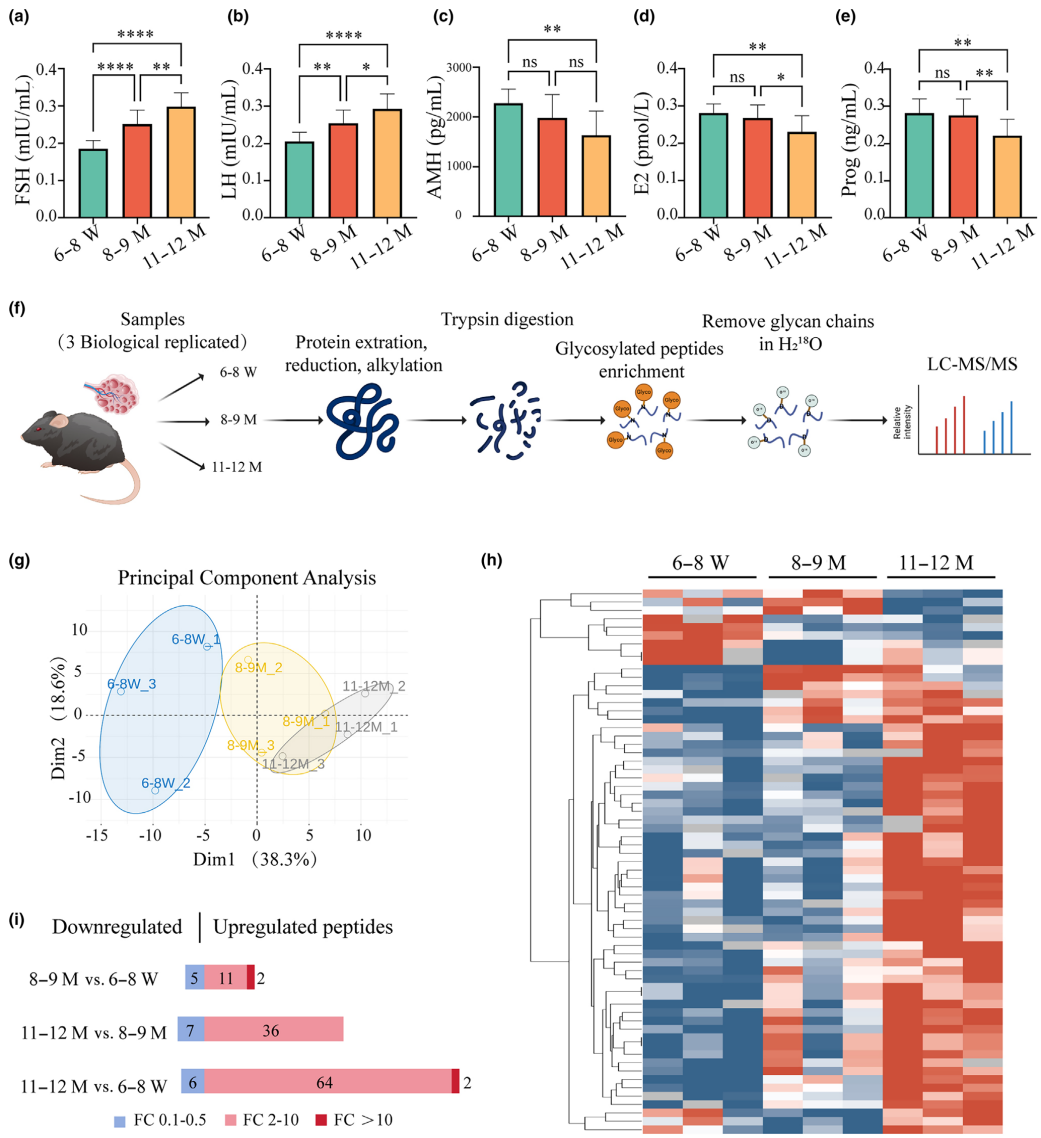
N‐glycoproteomics analysis in aging mice ovaries. (a–e) Graphical representation of hormone concentrations across three age groups with serum levels of FSH (a), LH (b), AMH (c), E2 (d), and Prog (e) quantified via ELISA. (f) Flowchart of the N‐glycoproteomic analysis process in ovaries from mice aged 6–8 W, 8–9 M, and 11–12 M. (g) PCA plot depicting the expression patterns of N‐glycopeptides among the three age groups. (h) Heatmap displaying the differential expression patterns of N‐glycopeptides across the three age groups. (i) Histogram representing the numbers of DE N‐glycopeptides, with increases shown in red and decreases in blue. Data in panels (a–e) are presented as mean ± SEM. AMH, anti‐Mullerian hormone; DE, differently expressed; E2, estradiol; FC, foldchange; FSH, follicle‐stimulating hormone; LH, luteinizing hormone; ns, not significant; PCA, principal component analysis; Prog, progesterone; 6–8 W, 6–8 weeks; 8–9 M, 8–9 months; 11–12 M, 11–12 months; *p < 0.05; **p < 0.01; ****p < 0.0001.

Furthermore, serum hormone levels were tested to determine the impact of aging on ovarian endocrine function. Significant increases in serum follicle‐stimulating hormone (FSH) and serum luteinizing hormone (LH) levels were recorded in the 8–9 M and 11–12 M groups when compared to the 6–8 W group (Figure 1a,b). In contrast, a substantial decline in AMH level was witnessed in the 11–12 M group when compared with the 6–8 W group (Figure 1c). Similarly, the serum levels of estradiol (E2) and Prog also exhibited notable decreases with aging (Figure 1d,e). These data implied a significant decline in reproductive function in female mice from the age of 8–9 M, which was comparable to the reproductive aging observed in 35‐year‐old human females (Boot et al., 1956; Coxworth & Hawkes, 2010), thereby highlighting the relevance of further investigations into the underlying biological events and molecular mechanisms during this pivotal period.

### 2.2 N‐glycoproteomic analysis of systematic variations in N‐glycoproteins during ovarian aging in mice

To unravel the variations in N‐glycosylated peptides in the ovaries during the aging of mice, an MS‐based quantitative N‐glycoproteomic approach was employed, as detailed in the flow chart (Figure 1f). A total of 1165 N‐glycopeptides and 622 N‐glycoproteins were quantified accordingly (Figure S2A). Venn diagrams illustrating the peptides and proteins among three biological replicates are presented in Figure S2B,C. It was noted that 936 glycopeptides and 524 glycoproteins were uncovered across the ovarian samples from 6–8 W, 8–9 M, and 11–12 M groups (Figure S2B,C). Principal component analysis (PCA) displayed that the expression patterns of the 8–9 M and 11–12 M groups were closely aligned when compared to the 6–8 W group (Figure 1g). The heatmap and volcano plot highlighted that the number of upregulated glycopeptides in the ovaries of mice aged 8–9 M and 11–12 M exceeded the number of downregulated ones (Figure 1h, S2D–F). Specifically, 13 upregulated glycopeptides and 5 downregulated ones were demonstrated in the ovaries of the 8–9 M group, and 66 upregulated glycopeptides and 6 downregulated ones were noted in the 11–12 M group when compared to the 6–8 W group (Figure 1i). Prominent examples of upregulated glycopeptides included Cpa3‐N242 and Gpnmb‐N200 in the 8–9 M versus 6–8 W comparison, and Lghm‐N280 and Lipg‐N80 in the 11–12 M versus 6–8 W comparison, each showing more than 10 fold increases. These data uncovered the significant role of increased N‐glycosylation levels and N‐glycoproteins in the process of ovarian aging.

Approximately 42.17% of glycoproteins had more than one glycosylation site (Figure S3A). Notably, Lrp1, a protein involved in regulating the ovarian microenvironment (Shen et al., 2024), was identified with 34 glycosylation sites. The expression changes of glycopeptides in ovaries during the aging process were categorized into nine types through fuzzy C‐means clustering (Figure S3B). Predominantly, glycopeptides, such as Lipg‐N80, Itgax‐N949, and Ctsl‐N221, exhibited a consistent upregulation in expression as aging progressed. Conversely, some glycopeptides were consistently downregulated, such as F12‐N415. Additionally, other glycopeptides demonstrated variable expression patterns; for example, Cpa3‐N242 was upregulated in the 8–9 M mice and subsequently downregulated in the 11–12 M mice (Figure S3C). Observations of multiple glycosylation sites in certain proteins revealed a variety of expression patterns at different sites in the same protein, including Pzp, Ighm, Lamp1, and Lamc1 (Figure S3C). These findings suggest that N‐glycosylated modifications at various glycosylation sites in the same protein might confer roles in various biological events.

**FIGURE 2:**
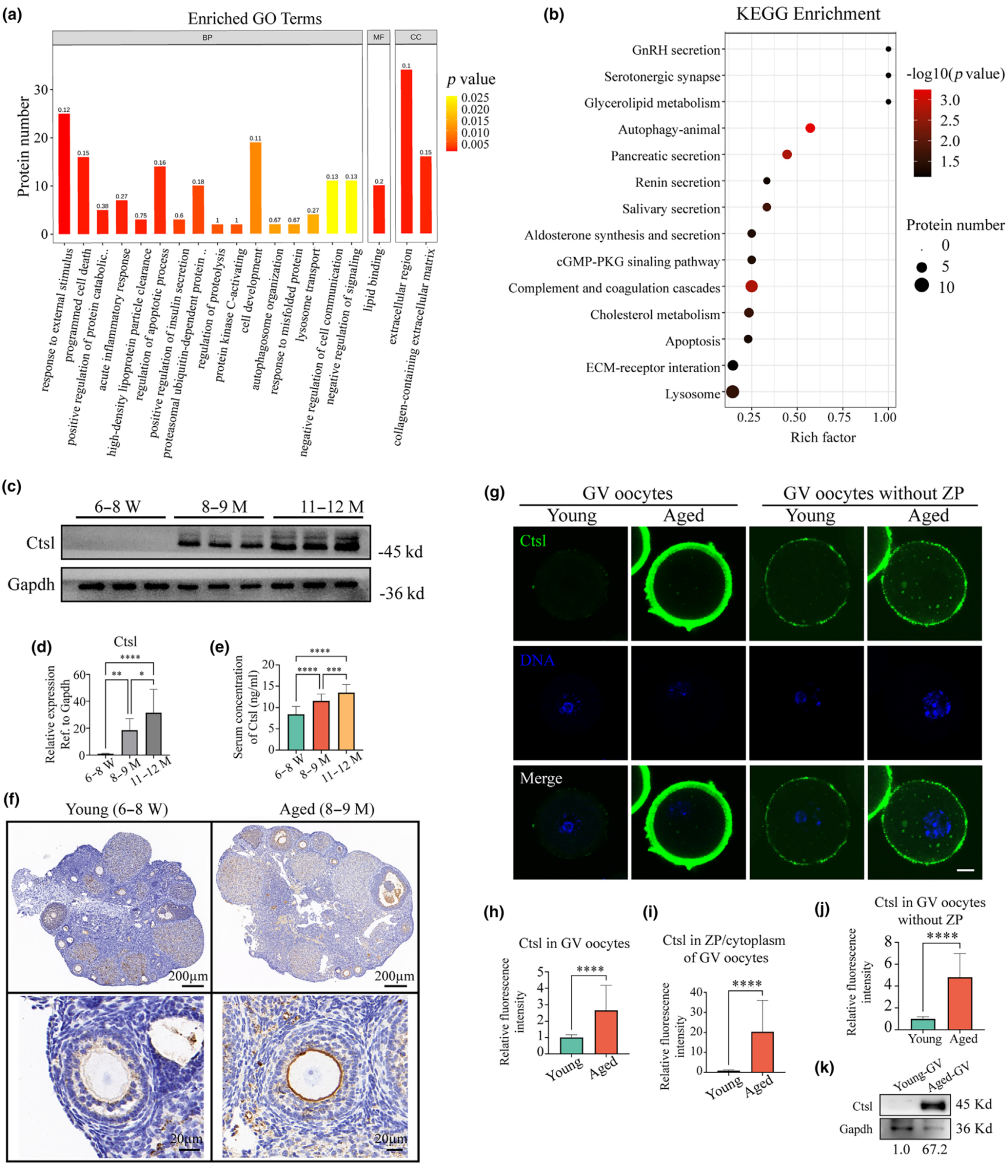
Enrichment analysis of N‐glycoproteomic data and Ctsl expression profiles in mouse ovarian aging. (a) Representative GO terms for the enrichment of DE N‐glycoproteins across the three age groups (6–8 W, 8–9 M, and 11–12 M). (b) Representative KEGG terms for the enrichment of DE N‐glycoproteins across the three age groups. (c) IB assay displaying the levels of Ctsl in ovaries of mice across three age groups. (d) Quantitative protein analysis of Ctsl across the three age groups. (e) Serum Ctsl concentrations in mice across the three age groups. Histochemistry of Ctsl in ovaries from young (6–8 W) and aged (8–9 M) mice. Brown, Ctsl; blue, nucleus. (f) Illustrations of Ctsl expression in young and aged GV oocytes with or without ZP. Scale bar, 10 μm. Green, Ctsl; blue, DNA. (g) FI comparison of Ctsl between young (*n* = 23) and aged (*n* = 23) GV oocytes. (h) ZP/Cytoplasm fluorescence ratios of Ctsl in young (*n* = 23) and aged (*n* = 23) GV oocytes. (i) FI of Ctsl signals without ZP in young (*n* = 24) and aged (*n* = 20) GV oocytes. IB analysis of Ctsl levels in young (*n* = 100) and aged (*n* = 100) GV oocytes. Data in panels (d), (e), (h), (i), and (j) are presented as mean ± SEM. FI, fluorescence intensity; GO, Gene Ontology; GV, germinal vesicle; IB, immunoblotting; KEGG, Kyoto Encyclopedia of Genes and Genomes; ZP, zona pellucida; *p < 0.05; **p < 0.01; ***p < 0.001; ****p < 0.0001.

DE N‐glycoproteins were further analyzed using gene ontology (GO) pathway annotation. The enrichment analysis revealed key processes implicated in aging, such as “response to external stimulus,” “programmed cell process,” “regulation of apoptotic process,” “regulation of proteolysis,” and “cell development” (Figure 2a). In addition, the Kyoto Encyclopedia of Genes and Genomes (KEGG) pathway analysis associated these DE N‐glycoproteins primarily with “autophagy‐animal,” “apoptosis,” and “lysosome” pathways (Figure 2b, S4A). Considering the established association between autophagic dysfunction and aging (Jin et al., 2022; Navarro‐Pando et al., 2021; Rubinsztein et al., 2011), subsequent analyses focused on the expression of autophagy‐related proteins (Figure S4B). Immunoblot analysis demonstrated a progressive increase in p62/Sqstm1 levels and a reduction in the ratio of microtubule‐associated protein 1 light chain 3 (Lc3b) II/I in the ovaries of 8–9 M and 11–12 M mice compared to those in the 6–8 W group, suggesting impaired autophagic function in the ovaries during aging (Figure S4B–D). Moreover, the protein levels of Lamp1, a classic marker of lysosomes, were evaluated across the groups (Figure S4B). The immunoblotting (IB) assay and semi‐quantitative assessments exhibited an elevation in Lamp1 levels in aged ovaries relative to younger mice, highlighting a potential involvement of lysosomal dysfunction in the aging process of the ovaries (Figure S4B,E).

### 2.3 Progressive increase of Ctsl expression in ovaries and oocytes during ovarian aging

To identify pivotal proteins involved in ovarian aging, the study focused on the analysis of DE N‐glycoproteins in the “lysosome,” “phagosome,” and “autophagy” pathways (Figure S4A,F). Protein–protein interaction (PPI) networks illustrated the interaction relationships among DE N‐glycoproteins in these pathways (Figure S4G). Among the 11 autophagy‐lysosome‐related N‐glycoproteins, Ctsl‐N221 demonstrated a 7.18‐fold upregulation in 11–12 M ovaries when compared to those in the 6–8 W group (Figure S4F). Volcano plots clearly further corroborated a gradual increase in the expression of Ctsl during the aging process (Figure S2D–F). Validation of these results through IB assay using an anti‐Ctsl antibody confirmed the upregulation (Figure 2c,d). Given the endocrine characteristics of N‐glycoproteins, the serum level of Ctsl was subsequently measured across the three groups, revealing a significant elevation with age (Figure 2e). This elevation pointed to the potential utility of Ctsl as a biomarker for assessing ovarian reserve. The expression of Ctsl was notably increased in the ovaries of mice aged 8–9 M, prompting the designation of 8–9 M mice as the aged group and the 6–8 W mice as the young group for further investigations.

Immunohistochemical staining unveiled that Ctsl expression was primarily localized in oocytes at different stages of folliculogenesis and in the corpus luteum, consistent with prior studies (Oksjoki et al., 2001). Additionally, Ctsl expression in the oocytes of aged mice was prominently higher than in young mice, with significant differences particularly evident in the zona pellucida (ZP) (Figure 2f). Further analysis using fluorescence intensity (FI) measurement showed a substantial increase in Ctsl signals of aged oocytes compared with their younger counterparts, especially in the ZP region (Figure 2g–i). To minimize the interference from high fluorescence signals in the ZP region, the expression of Ctsl in the cytoplasm of oocytes without ZP was compared between young and aged oocytes using Tyrode's solution. The results indicated a significant increase in Ctsl expression in aged oocytes than those in young ones (Figure 2g,j). These observations were corroborated by immunoblot analysis comparing Ctsl protein levels in germinal vesicle (GV) oocytes from young and aged mice, which confirmed the increased Ctsl protein levels in aged oocytes (Figure 2k). The same trend was observed in metaphase II (MII) oocytes, validated through immunofluorescence (IF) staining and IB analysis (Figure S5A–E). Collectively, these findings established that Ctsl expression was markedly elevated not only in the ovarian tissue but also in oocytes during the aging process.

### 2.4 Impact of Ctsl overexpression and N‐glycosylation at the Ctsl‐N221 site on meiotic maturation

**FIGURE 3:**
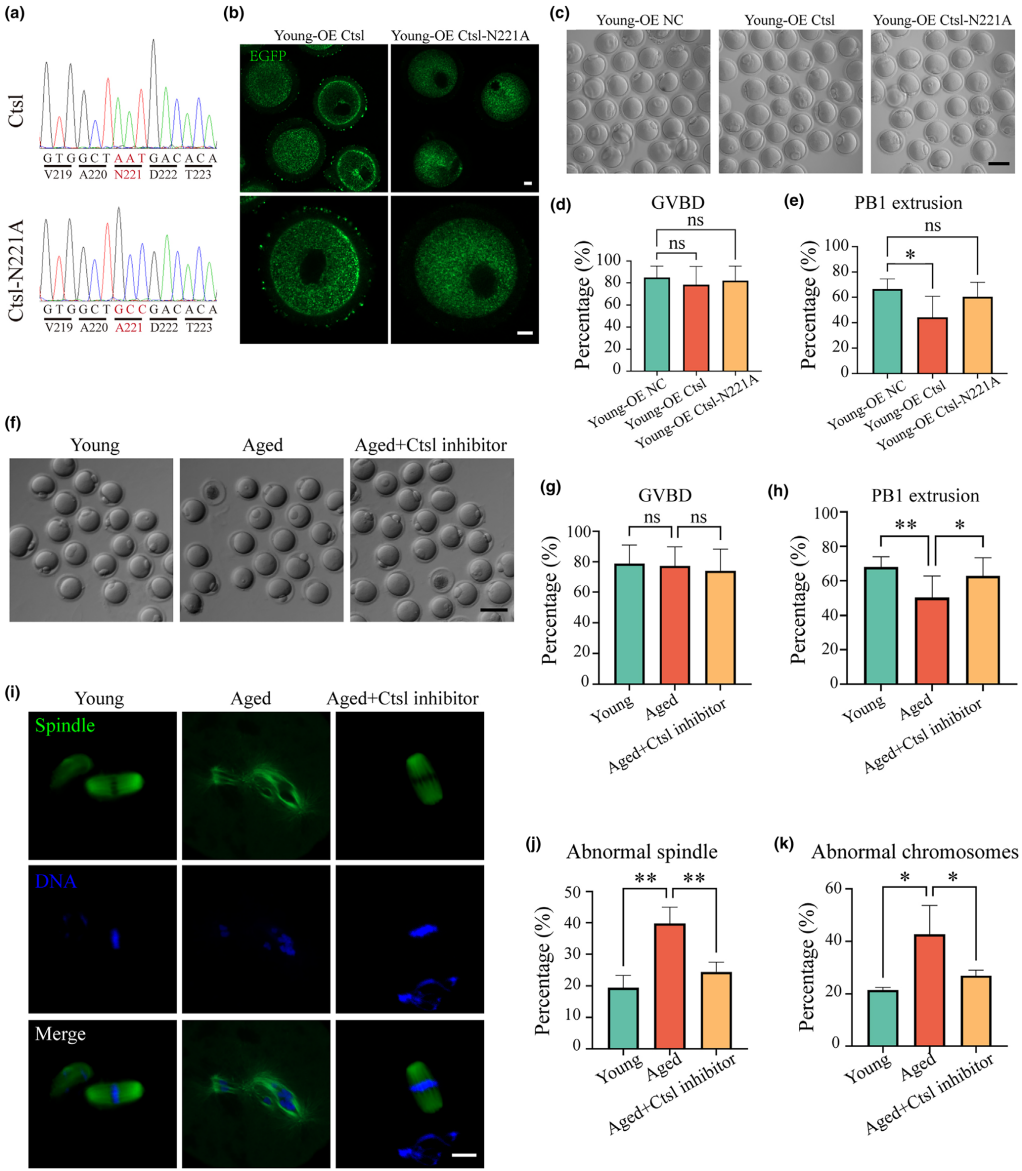
Overexpression of Ctsl in young oocytes and Ctsl inhibitor supplementation in aged oocytes. (a) The oligonucleotide sequences of Ctsl and Ctsl‐N221A mutation for OE experiment. (b) Localization of synthesized Ctsl in young‐OE Ctsl and young‐OE Ctsl‐N221 groups. Scale bar, 10 μm. (c) Representative images depicting oocytes cultured in vitro from three experimental conditions: young‐OE NC (*n* = 89), young‐OE Ctsl (*n* = 145), and young‐OE Ctsl‐N221A (*n* = 103) oocytes. Scale bar, 100 μm. GVBD rates across the three age groups. PB1 extrusion rates across the three age groups. (d) Representative images of oocytes cultured in vitro from three groups: young (*n* = 296), aged (*n* = 171), and aged+Ctsl inhibitor (*n* = 185). Scale bar, 100 μm. (e) GVBD rates in young, aged, and aged+Ctsl inhibitor‐treated oocytes. (f) PB1 extrusion rates in the three experimental groups. (g) Visualization of spindle morphology and chromosome alignment at MII in the three experimental groups: young (*n* = 72), aged (*n* = 65), and aged+Ctsl inhibitor (*n* = 60). Spindles and chromosomes were visualized using immunostaining with ɑ‐tubulin‐FITC antibody and Hoechst 33342, correspondingly. Scale bar, 10 μm. Green, spindle; blue, DNA. (h) Abnormal spindle rates in the three experimental groups. Misaligned chromosome rates in the three experimental groups. Data in panels (d), (e), (g), (h), (j) and (k) are presented as mean percentages (mean ± SEM). Ctsl‐N221A, N221A‐mutated‐Ctsl; GVBD, germinal vesicle breakdown; NC, negative control; ns, not significant; OE, overexpression; PB1, first polar body. *p < 0.05; **p < 0.01.

Given the elevated levels of Ctsl in aged oocytes, it was hypothesized that overexpression (OE) of Ctsl might mediate the quality of young oocytes. Initially, a plasmid encoding Ctsl tagged with EGFP at its C‐terminus was constructed. Subsequently, cRNA for Ctsl‐EGFP was microinjected into the cytoplasm of young oocytes at GV‐stage, which were then cultured in M16 medium with milrinone for 4–6 h to facilitate Ctsl‐EGFP protein synthesis. The oocytes were transferred to fresh M16 medium to continue meiotic maturation. In light of the substantial increase in Ctsl‐N221 glycopeptides in aged ovaries (Figure S4F), it was speculated that the N221 site of Ctsl was a critical glycosylation site. Therefore, we simultanously microinjected cRNA encoding the N221A‐mutated Ctsl into GV oocytes to examine the process of oocyte maturation (Figure 3a).

The distribution of newly synthesized Ctsl protein tagged with EGFP was tracked to assess its localization in oocytes. In the OE‐Ctsl group, the green fluorescent signals emanating from the EGFP tag were present not only in the cytoplasm but also in the ZP of the oocytes. Conversely, these signals of EGFP in OE‐Ctsl‐N221A group were confined to the cytoplasm of oocytes (Figure 3b). The distinct localization patterns indicated that the N‐glycosylation at the Ctsl‐N221 site facilitated the translocation of Ctsl protein to the ZP of oocytes.

Germinal vesicle breakdown (GVBD) rates and first polar body (PB1) extrusion rates were subsequently calculated (Figure 3c). The analysis revealed no significant differences in GVBD rates across the three groups: OE NC, 84.88%; OE Ctsl, 78.29%; and OE Ctsl‐N221A, 82.00%; *p* > 0.05 (Figure 3d). Nevertheless, the PB1 extrusion rate in the OE‐Ctsl group was markedly lowered relative to the control group (44.36% vs. 66.55%, *p* < 0.05), while it remained similar between the OE‐Ctsl‐N221A group and the control (60.42% vs. 66.55%, *p* > 0.05) (Figure 3e). This differential impact confirmed that overexpression of Ctsl disrupted meiotic maturation, whereas the missense mutation at the N221 site mitigated this effect, underscoring the significance of N‐glycosylation at this site as a critical PTM.

### 2.5 Ctsl inhibitor supplementation restores meiotic maturation in aged oocytes

The impact of Ctsl inhibition on oocyte maturation was investigated by supplementing the in vitro culture system with a Ctsl inhibitor, dosed according to guidelines from prior research (Nofal et al., 2022). GV oocytes isolated from young and aged mice were cultured to evaluate their maturation potential. For the aged oocytes, the culture medium was enhanced with the Ctsl inhibitor. It was observed that the PB1 extrusion rate was 50.30% in aged oocytes, significantly lower than 67.98% in young oocytes; however, this deficiency was counteracted upon the introduction of the Ctsl inhibitor into the culture of aged oocytes (62.83%) (Figure 3f–h).

Further analysis focused on the structural integrity of meiotic spindles and chromosome alignment, known to be compromised in aged oocytes (Baird et al., 2005). Abnormalities in spindle organization and chromosome alignment were quantified using IF staining (Figure 3i). The quantitative analysis revealed that disorganized spindle apparatuses and misaligned chromosomes were significantly higher in aged oocytes compared to young oocytes, which could be mitigated by the application of the Ctsl inhibitor (Figure 3j,k).

### 2.6 Ctsl inhibitor supplementation enhances mitochondrial function and mitigates cellular anomalies in aged oocytes

**FIGURE 4:**
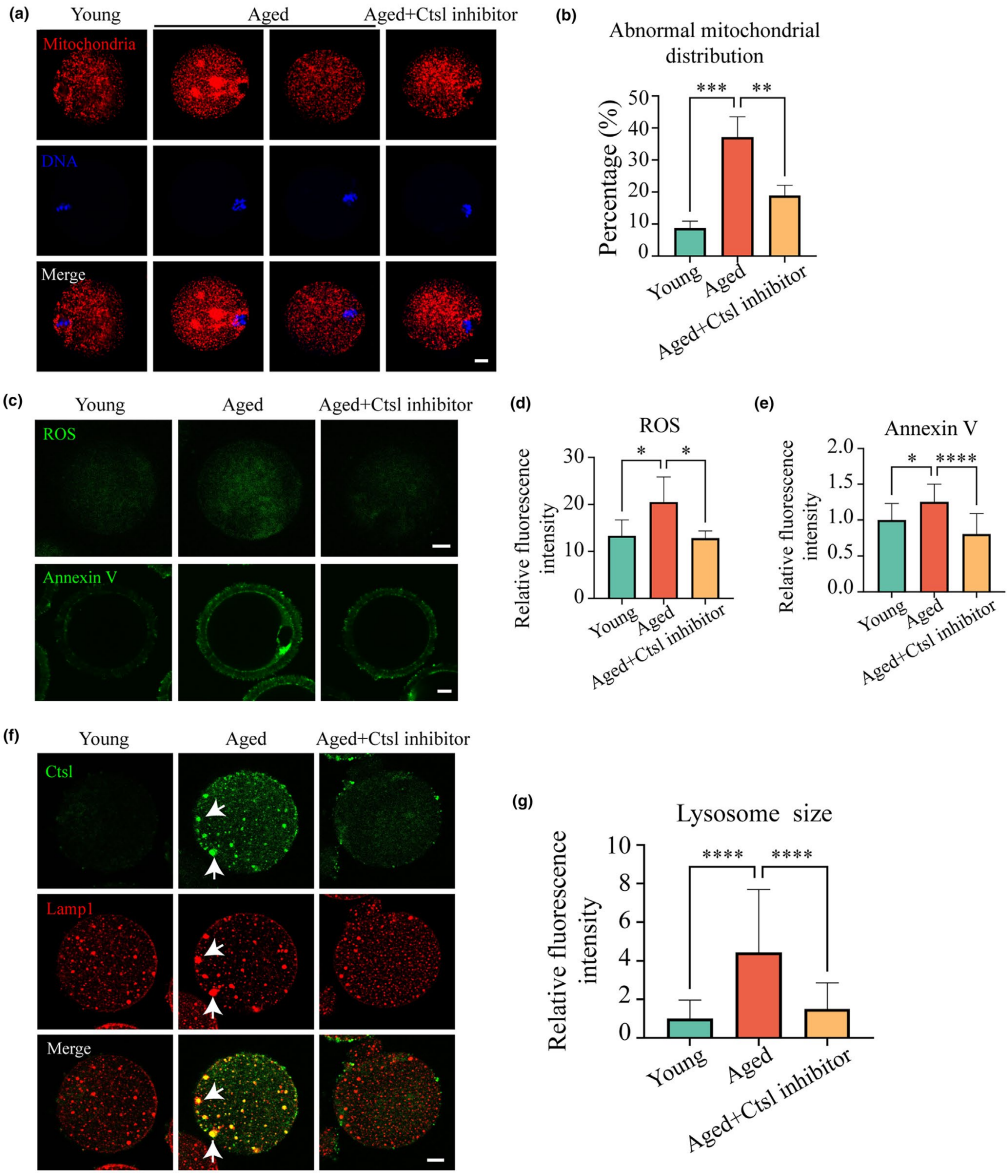
Impact of Ctsl inhibitor supplementation on oocyte quality in aged mice. Visualization of mitochondrial distribution in the three experimental groups. Mitochondria and chromosomes were visualized using MitoTracker staining and Hoechst 33342. Scale bar, 10 μm. Red, mitochondria; blue, DNA. (a) Abnormal mitochondrial distribution rates among the three experimental groups: young (*n* = 36), aged (*n* = 32), and aged+Ctsl inhibitor (*n* = 37). Detection of ROS accumulation and apoptosis using DCFH staining and Annexin V staining in the three experimental groups. ROS generation was evaluated. Scale bar, 10 μm. (b) FI of ROS signals in the three experimental groups: young (*n* = 35), aged (*n* = 30), and aged+Ctsl inhibitor (*n* = 32). (c) FI of Annexin V in the three experimental groups: young (*n* = 36), aged (*n* = 29), and aged+Ctsl inhibitor (*n* = 28). (d) Colocalization of Ctsl and Lamp1 in the three experimental groups. Scale bar, 10 μm. Green, Ctsl; red, Lamp1; yellow, white arrows indicate colocalization of Ctsl and Lamp1. (e) Lysosome size measurement in the three experimental groups based on the area of Lamp1 signals. Data in panels (b) are presented as mean percentages (mean ± SEM); Data in panels (d), (e), and (g) are presented as mean ± SEM. ROS, reactive oxygen species; *p < 0.05; **p < 0.01; ***p < 0.001; ****p < 0.0001.

Mitochondrial function is a critical factor in determining oocyte quality; thus, the distribution of mitochondria was assessed in young, aged, and Ctsl inhibitor‐treated aged oocytes using MitoTracker staining. Normal mitochondrial distribution, characterized by an even dispersal in the cytoplasm and accumulation around the periphery of chromosomes, was observed in the majority of the young oocytes, aligning with findings from previous studies (Miao et al., 2020). In contrast, many aged oocytes exhibited a loss of peripheral accumulation and an aggregated distribution within the cytoplasm (Figure 4a). The proportion of aged oocytes with abnormal mitochondrial distributions was quantitatively measured at over 37.2%, a marked contrast to the 8.8% found in young oocytes. Furthermore, this aberration was prominently reduced to 18.9% after treatment with the Ctsl inhibitor (Figure 4b). Hence, the results demonstrated that the mislocalization of mitochondria in aged oocytes could be ameliorated through a Ctsl inhibitor.

The impact of Ctsl inhibition on mitochondrial membrane potential (ΔΨm, MMP) was evaluated in young, aged, and Ctsl inhibitor‐treated aged oocytes. High MMP was indicated by red fluorescence, whereas low MMP was marked by green fluorescence, discernible through JC‐1 staining. The analysis demonstrated a notable reduction in the red‐to‐green fluorescence ratio in aged oocytes relative to their young counterparts, an imbalance that was partially rectified in aged oocytes following Ctsl inhibitor treatment (Figure S6A,B). These observations indicated the potential of Ctsl inhibition to reverse age‐related mitochondrial impairment in oocytes.

Given the established relationship between mitochondrial dysfunction and the generation of ROS, which in turn can further impair mitochondrial function (Cao et al., 2022; van der Reest et al., 2021), ROS levels were subsequently measured using DCFH‐DA staining. Interestingly, ROS signals were more intense in aged oocytes than in young oocytes, but these elevated levels were alleviated in Ctsl inhibitor‐treated aged oocytes, bringing them on par with those observed in young oocytes (Figure 4c,d). Collectively, these results affirmed that Ctsl inhibitor supplementation relieved mitochondrial dysfunction and reduced ROS accumulation in aged oocytes.

Apoptosis in young, aged, and Ctsl inhibitor‐treated aged oocytes was evaluated using Annexin‐V staining. This analysis uncovered that the increased apoptosis observed in aged oocytes could be mitigated by the application of the Ctsl inhibitor (Figure 4c,e). Further investigations into the molecular basis of this observation were conducted via RT‐PCR to measure the expression levels of key genes involved in apoptosis (*Bax* and *p53*) and autophagy (*Lc3b* and *Becn‐1*). These studies confirmed that the expression abnormalities in *Bax* and *Lc3b* in aged oocytes were restored to levels observed in young oocytes by the Ctsl inhibitor (Figure S6C–F).

In addition, interactions between Ctsl and Lamp1, a classical lysosomal marker, were analyzed through PPI studies, which revealed interactions particularly pronounced in aged oocytes (Figure S4G). The localization of Ctsl and Lamp1 was subsequently examined in the three study groups. IF staining further detailed the colocalization patterns of Ctsl and Lamp1, which were significantly altered in the aged oocytes compared to the other groups (Figure 4f). Notably, the aged group displayed an increase in lysosome size, an indicator of potential lysosomal dysfunction (Figure 4g). Together, these findings suggest that treatment with a Ctsl inhibitor not only curtails apoptotic signaling and restores lysosomal size and function but also reestablishes a healthier cellular environment in aged oocytes.

### 2.7 Ctsl KD promotes meiotic maturation and embryonic development in aged oocytes

**FIGURE 5:**
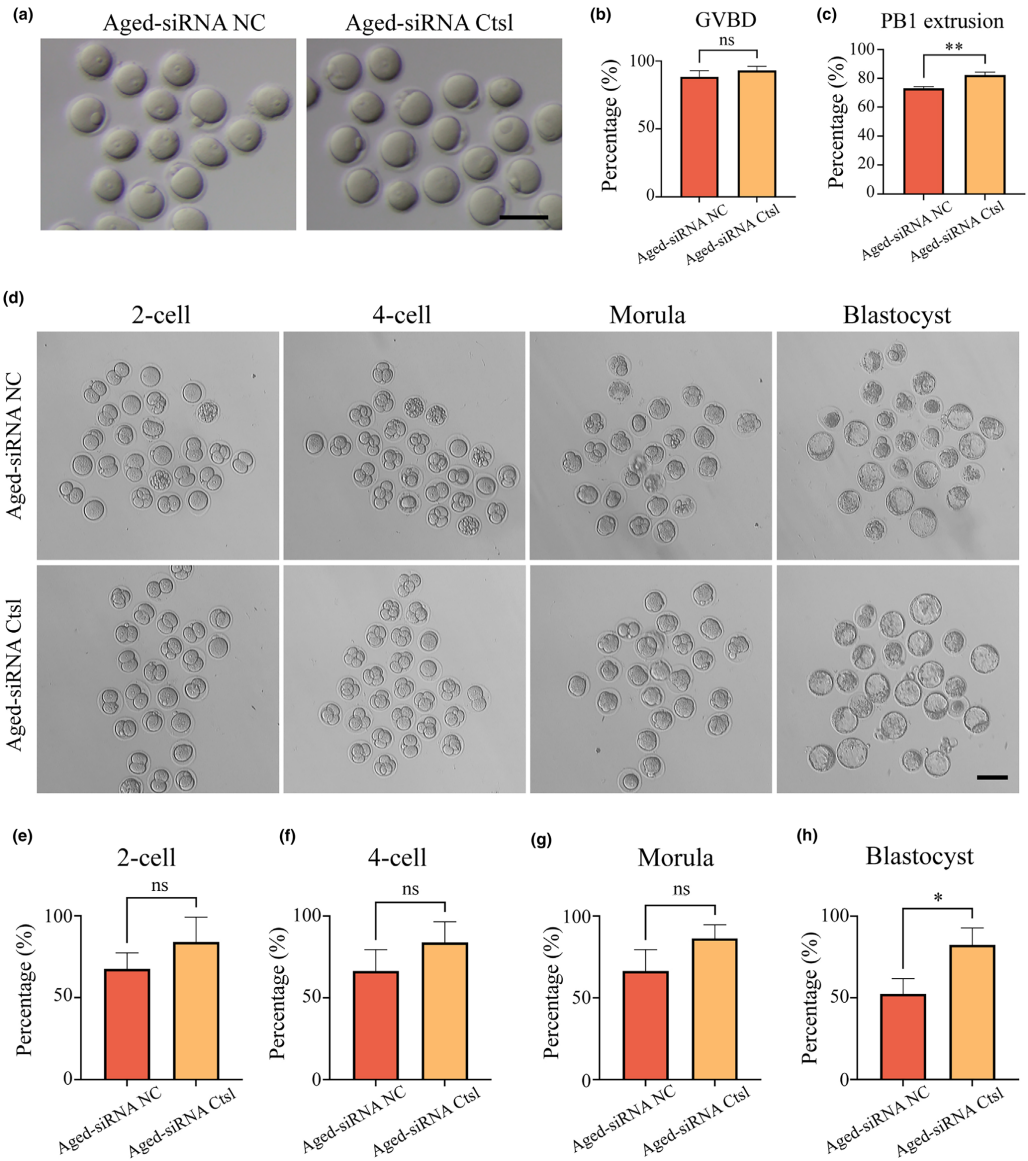
Knockdown Ctsl in aged oocytes restored oocyte quality and enhanced embryo developmental potential. (a) Illustration of in vitro cultured oocytes from the aged‐siRNA NC (*n* = 131) and aged‐siRNA Ctsl (*n* = 111) groups. Scale bar, 100 μm. (b) GVBD rates in oocytes of the aged‐siRNA NC and aged‐siRNA Ctsl groups. (c) PB1 extrusion rates in oocytes of the aged‐siRNA NC and aged‐siRNA Ctsl groups. (d) Representative images of early embryos from oocytes of the aged‐siRNA NC (*n* = 129) and aged‐siRNA Ctsl (*n* = 152) groups. Scale bar, 100 μm. (e) Fertilization rates in oocytes of the aged‐siRNA NC and aged‐siRNA Ctsl groups. (f) Rate of 4‐cell embryos in oocytes of the aged‐siRNA NC and aged‐siRNA Ctsl groups. (g) Morula rates in oocytes of the aged‐siRNA NC and aged‐siRNA Ctsl groups. (h) Blastocyst formation rates in oocytes of the aged‐siRNA NC and aged‐siRNA Ctsl groups. Data in panels (b), (c), (e–f) are presented as mean percentages (mean ± SEM). siRNA, small interfering RNA; ns, not significant; *p < 0.05; **p < 0.01.

The function of Ctsl during oocyte maturation was further investigated by knocking down its expression. Ctsl expression in aged GV oocytes was reduced through microinjection of small interfering RNA (siRNA) targeting Ctsl, while control aged oocytes were concurrently microinjected with negative control siRNA (siRNA‐NC). Following injection, the oocytes were cultured in an M16 medium containing milrinone for 24 h to arrest development and subsequently transferred to a fresh M16 medium to resume the meiotic process. The rates of GVBD and PB1 extrusion were then measured. The findings revealed an enhancement in PB1 extrusion rates in the aged oocytes treated with siRNA‐Ctsl (aged+siRNA Ctsl) compared to those in the control group (Figure 5a–c). This improvement signified that Ctsl KD fostered the meiotic maturation and potentially the embryonic development potential of aged oocytes.

In addition, the expression of Ctsl was compared between GV and MII oocytes in young mice through an IF assay (Figure S7A). The quantitative findings demonstrated higher Ctsl protein levels in GV oocytes than in MII oocytes (Figure S7B). Additional assessments using RT‐PCR and IB results displayed prominently diminished mRNA and protein levels in MII oocytes relative to GV oocytes (Figure S7C,D). This observation led to the hypothesis that high expression of Ctsl in the MII oocytes of aged mice might impair fertilization capacity and embryonic developmental potential. Thus, Ctsl expression was knocked down in aged MII oocytes through siRNA‐Ctsl injection, followed by evaluation of their fertilization capacity via in vitro fertilization (IVF) experiments. Although more oocytes from aged mice were fertilized and progressed to 2‐cell embryos in response to Ctsl KD, the difference was not statistically significant (Figure 5d,e). Subsequent stages, including rates of 4‐cell embryos, morula, and blastocyst, were higher in the siRNA‐Ctsl group compared to control groups (Figure 5d,f–h), with the difference in blastocyst rate reaching statistical significance (Figure 5h). Overall, these data indicated the potential of Ctsl KD to improve the reproductive success of aged oocytes by facilitating better oocyte quality, fertilization, and early embryonic development.

## 3 DISCUSSION

Age is universally acknowledged as an independent factor affecting female reproductive function (Crawford & Steiner, 2015; Gruhn et al., 2019). In contemporary society, the postponement of childbearing has emerged as a noteworthy issue, exacerbating age‐related challenges in fertility and increasing the risk of adverse pregnancy outcomes (Johnson et al., 2012; Sauer, 2015). Despite extensive research, the molecular mechanisms underlying the decline in ovarian function as age progresses remain not fully elucidated.

In this research, we established that the ovarian function of C57BL/6 female mice declined with aging, as evidenced by follicle counts and serum hormone assays, which are consistent with a previous report (Sauer, 2015). This validation led us to utilize the natural aging model in mice to delve deeper into the molecular processes driving ovarian senescence.

N‐glycosylation, as a crucial PTM in ovarian physiology, imparts vital effects on ovarian function by modifying a variety of N‐glycoproteins, such as hormones, receptors, and growth factors, to modulate ovarian function (Bousfield & Harvey, 2019; Pankhurst & McLennan, 2013). Nevertheless, detailed insights into N‐glycosylated modifications in ovarian tissues remain sparse. Our research aimed to fill this gap by systematically exploring the alterations in N‐glycosylated proteins during ovarian aging. Proteomic analyses indicated an upregulation in the number of glycopeptides in the ovaries of reproductively aged mice, with only a few being downregulated. Thus, we hypothesized that high levels of N‐glycosylated proteins and an increase in N‐glycoproteins might contribute to ovarian failure and oocyte senescence. Further bioinformatics analysis revealed that DE N‐glycoproteins were predominantly enriched in pathways associated with lysosomes, phagosomes, and autophagy. Autophagy dysfunction and lysosomal perturbations have been implicated in the progression of aging and related pathologies (Kaushik et al., 2021; Kumariya et al., 2021; Leeman et al., 2018). Our findings from IB analyses demonstrated an increase in p62 expression and a decrease in the Lc3b II/I ratio in ovaries of mice from 8 to 9 M, consistent with reductions in autophagic efficiency noted in prior research (Navarro‐Pando et al., 2021). Additionally, an increase in Lamp1 protein level was observed with ovarian aging. These results highlight the potential blockage of autophagy‐lysosomal flux as a critical factor in the deterioration of ovarian function. Further investigations are underway to identify the key glycoproteins driving these pathophysiological changes.

Through comparative analysis of DE N‐glycoproteins, particular attention was given to Ctsl, which exhibited a marked upregulation during the aging process. Ctsl belongs to the cysteine protease family, which is predominantly upregulated in disease states and implicated in multiple pathological conditions (Yadati et al., 2020). Prior research has linked elevated Ctsl levels to neurotoxicity and its involvement in the pathogenesis of Parkinson's disease (Jiang et al., 2022), a neurodegenerative disorder closely related to aging. Furthermore, research has demonstrated that the deletion of Ctsl restricts the severity of pancreatitis in mice, and its inhibition is considered to confer therapeutic benefits (Wartmann et al., 2010). However, the implications of Ctsl in ovarian failure and oocyte senescence remain largely unexplored. In our research, we identified the expression of Ctsl in ovaries, serum, and oocytes, noting that a progressive increase in serum Ctsl levels in female mice as they aged. This observation underscores the potential of Ctsl as an indicative biomarker of ovarian aging in clinical settings.

The C57BL/6 female mouse model is extensively utilized for studying the aging process in female reproductive systems due to its similar patterns of changes in ovarian structure and function that parallel those observed in human aging. The age bracket of 8–9 M in mice, analogous to 35‐year‐old human females (Coxworth & Hawkes, 2010), served as an appropriate model for exploring the molecular underpinnings of ovarian senescence. Our study focused particularly on this age group. We uncovered a pronounced increase in Ctsl expression in aged oocytes, especially in the ZP of oocytes. Subsequently, we experimentally increased the expression of Ctsl in young oocytes through microinjection of its mRNA and subsequently assessed their maturation rates in vitro. The data unveiled that the overexpression of Ctsl in young oocytes impeded meiotic maturation, confirming its detrimental role in maintaining oocyte quality. Interestingly, when the N221 glycosylation site of Ctsl was mutated to disrupt its N‐glycosylated modification, the detrimental effects previously observed were not replicated. While exogenous Ctsl localized to the cytoplasm and ZP of oocytes, the N221A‐mutant Ctsl was only found in the cytoplasm and did not exhibit exocytosis functionality, highlighting that the N221 glycosylation modification is crucial for its activity.

To delineate the role of Ctsl in oocytes, its function was disrupted in aged oocytes, and the impact on meiotic maturation was assessed in vitro using selective inhibitors and KD approaches. The supplementation of a Ctsl inhibitor and the KD of Ctsl expression were found to enhance the maturation of aged oocytes in vitro. Additionally, inhibiting Ctsl in aged oocytes facilitated nuclear maturation and recovered the spindle/chromosome structure. Mitochondrial function, a key indicator of oocyte quality, often deteriorates with age (May‐Panloup et al., 2016). The current study also demonstrated that Ctsl inhibitor supplementation could ameliorate mitochondrial dysfunction, eliminate ROS accumulation, and decrease apoptosis in aged oocytes. Furthermore, the potential of oocytes to undergo successful fertilization and subsequent embryonic development serves as a critical measure of oocyte quality (Conti & Franciosi, 2018). In this context, our experiments revealed that the KD of Ctsl in aged MII oocytes led to an increased rate of blastocyst formation, suggesting that high expression of Ctsl might compromise oocyte quality.

**FIGURE 6:**
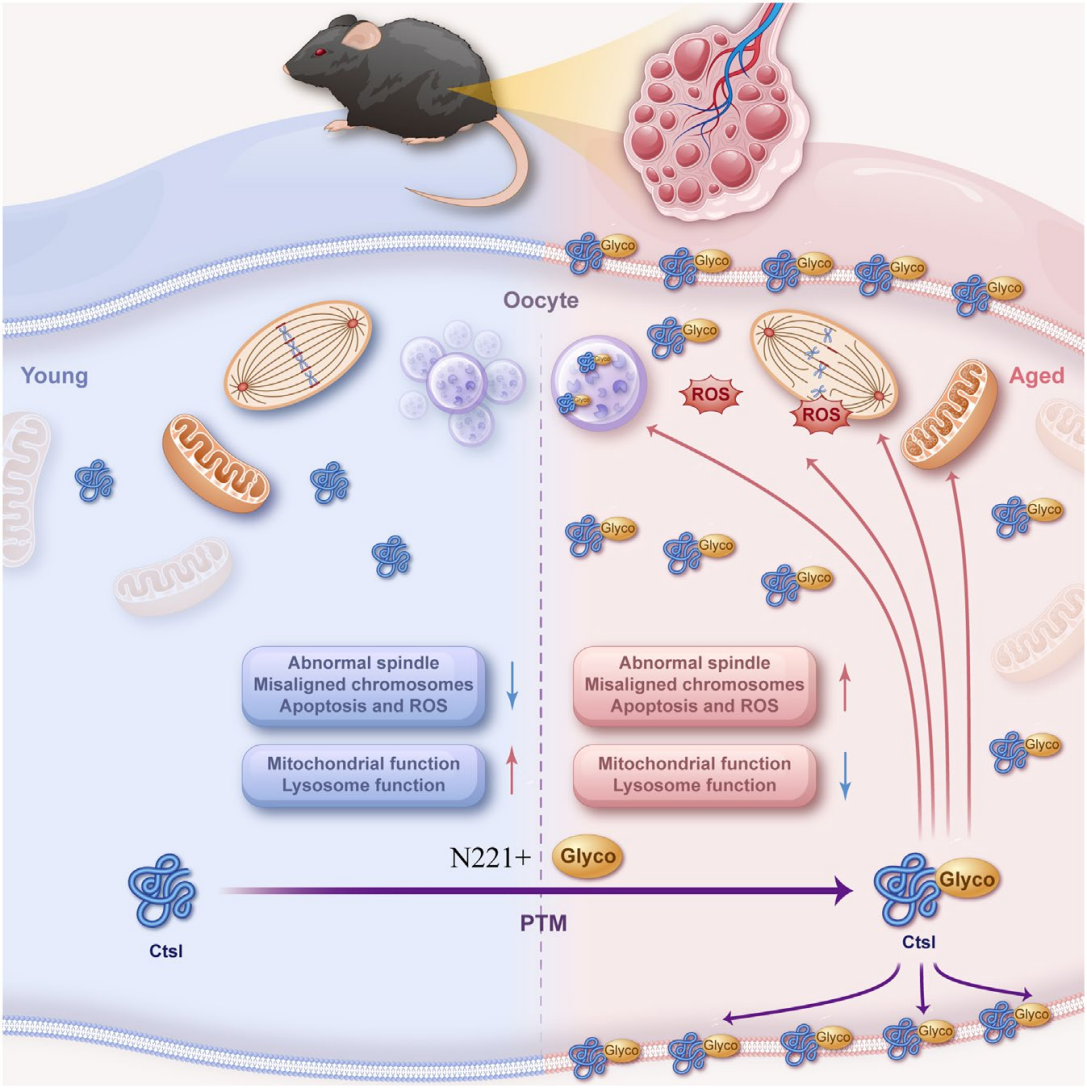
Schematic illustration of proposed mechanisms by which high expression of N‐glycosylated Ctsl impairs oocyte function and contributes to oocyte senescence. Ctsl protein is synthesized in the oocyte cytoplasm, where the N221 site undergoes N‐glycosylation. During oocyte aging, more glycosylated Ctsl proteins are generated and secreted through ZP into the extracellular matrix to confer their effects. Excessive Ctsl in the cytoplasm can damage mitochondrial function and generate ROS. Furthermore, Ctsl can accumulate in lysosomes and block the autophagy‐lysosome flux, which in turn elevates apoptotic potential, disrupts spindle formation and chromosome alignment, and hampers meiotic maturation. These combined effects ultimately reduce fertilization capacity and embryo developmental potential.

We propose the following hypothesis based on the findings presented (Figure 6). Ctsl protein is synthesized in the oocyte cytoplasm, undergoing N‐glycosylation at its N221 site. In the oocyte aging process, an increase in the production and secretion of glycosylated Ctsl proteins occurs, with these proteins moving through the ZP into the extracellular matrix to fulfill their functional roles. Excessive intracytoplasmic Ctsl can impair mitochondrial function and lead to increased production of ROS. This rise in ROS accumulation exacerbates mitochondrial dysfunction. Moreover, the accumulation of N‐glycosylated Ctsl in lysosomes may hinder the autophagy‐lysosome flux, compromise lysosomal function, increase apoptosis, and disrupt spindle formation and chromosome alignment, thereby adversely affecting meiotic maturation. These alterations ultimately influence the capacity for fertilization and embryonic development. A noted limitation of this research is its primary concentration on the role of Ctsl in oocytes, with its impact on the extracellular matrix still to be thoroughly explored.

In conclusion, our findings clarify the pivotal role of Ctsl in the aging process of oocytes, specifically emphasizing the essential role of N‐glycosylation at the Ctsl‐N221 site. The serum levels of Ctsl may be indicative of ovarian reserve, suggesting its potential as a clinical biomarker. Furthermore, the Ctsl protein and its glycosylated N221 site present promising targets for strategies for delaying ovarian failure and restoring oocyte quality in reproductive aging.

## 4 MATERIALS AND METHODS

### 4.1 Experimental animal

C57BL/6 mice were acquired from Nanjing Medical University, with all aspects of their care and use being compliant with the guidelines approved by the Institutional Animal Care and Use Committee of Nanjing Medical University (Approval number: IACUS‐2404108). The mice were housed under optimal conditions, including a stable temperature (20–24°C) and a consistent light–dark cycle (12 h light/12 h dark). They received humane treatment and unrestricted access to food and water. Mice were sacrificed by cervical dislocation. The ovaries and oviducts of female mice and epididymides of male mice were separated according to different experiments.

### 4.2 Oocyte collection, in vitro maturation, and IVF

GV oocytes were harvested from the ovaries and initially cultured in M2 medium (M7161, Sigma‐Aldrich, USA). Subsequently, they were transferred to M16 medium (M7292, Sigma‐Aldrich) covered with mineral oil (ART‐4008P, SAGE, USA) and maintained at 37°C in a 5% CO_2_ atmosphere for 16 h. Ctsl inhibitor (sc‐3132, Santa Cruz Biotechnology) was supplemented to the M16 medium at a concentration of 1.5 μM, as referenced in a prior study (Nofal et al., 2022).

MII oocytes were collected following hormonal induction with 10 IU of pregnant mare serum gonadotropin (PMSG) for 46–48 h, succeeded by 10 IU of human chorionic gonadotropin (hCG). After 14–16 h, cumulus‐oocyte‐complexes (COCs) were retrieved from the oviductal ampullae and placed in the M2 medium. Cumulus cells surrounding the oocytes were removed using 1 mg/mL hyaluronidase (H3884, Sigma‐Aldrich), and only matured oocytes exhibiting the PB1 were selected for subsequent procedures.

For IVF, spermatozoa were extracted from the epididymides of male mice and pre‐incubated in HTF medium (ART‐1020, SAGE, USA) under mineral oil at 37°C with 5% CO_2_ for 1 h. The prepared MII oocytes were then inseminated with the capacitated spermatozoa for 4 h. Following fertilization, the embryos were washed and cultured in KSOM medium (MR‐121‐D, Sigma‐Aldrich) to facilitate embryo development studies.

### 4.3 Microinjection of siRNA/mRNA and in vitro maturation

Microinjection was employed to knock down and overexpress Ctsl expression in oocytes through microinjection according to established methodologies from earlier research (Lin et al., 2022).

Ctsl expression was knocked down by cytoplasm microinjection of siRNA sequence. Specifically, Ctsl KD was achieved through the cytoplasmic microinjection of siRNA sequences in oocytes (Table S2). A nonspecific siRNA from RiboBio (Guangzhou, China) served as an NC. The procedure involved the introduction of 10 pL of siRNA solution (20 μM) with a micromanipulator system (IM‐300, Narishige, Japan) into GV stage oocytes suspended in M2 medium supplemented with 1 μM milrinone (M4655, Sigma‐Aldrich). Following injection, the GV oocytes were maintained in an M2 medium with milrinone for 24 h to allow for the KD of Ctsl expression. Thereafter, the oocytes were rinsed with milrinone‐free M2 medium and further cultured in M16 medium under mineral oil at 37°C in a 5% CO_2_ atmosphere. MII oocytes were microinjected and allowed to be cultured for an additional 2 h in an M2 medium for further IVF studies.

The full‐length *Ctsl* was integrated into the pcDNA3 vector (H341, OBiO Technology, Shanghai, China), which included an *EGFP* tag at the C‐terminal for visualization. To explore the specific contributions of glycosylation to Ctsl protein function, site‐directed mutagenesis was conducted at the N221 glycosylation site. The AAT to GCC transition at nucleotide 661–663 of the coding region caused missense mutation of the 211th aspartic acid (N) into alanine (A), hereafter referred to as Ctsl‐N221A mutation.

The plasmids were prepared for mRNA synthesis by linearization using the restriction enzyme Xho I (1094S, Takara, Japan). In vitro transcription of mRNA was achieved using the HiScribe® T7 ARCA mRNA Kit (E2060S, NEB, USA). In the subsequent overexpression experiments, mRNA (500 ng/μL) was delivered into GV stage oocytes via a 10 μL microinjection. These oocytes were initially arrested in an M2 medium supplemented with 1 μM milrinone for 4 h, after which they were transferred to an M16 medium to resume meiosis.

### 4.4 Statistical analysis

The experimental procedures were replicated at least three times, except where specifically noted. The statistical difference for two‐group comparisons was determined using an unpaired Student's *t* test. One‐way ANOVA was applied for evaluations involving multiple groups. All statistical computations were carried out using GraphPad Prism version 8. Results were expressed as the mean ± standard error of the mean (SEM). A *p* value threshold of less than 0.05 was set to determine statistical significance. In graphical representations, “ns” denoted nonsignificance, whereas “*p < 0.05”, “**p < 0.01”, and “***p < 0.001” indicated increasing levels of significance.

## AUTHOR CONTRIBUTIONS

KZ, BY, RM, and LC conceptualized and designed the research study. KZ, RX, LZ, HZ, ZQ, CL, ZH, and ZL conducted the experiments. KZ, RX, and JM were responsible for data analysis. KZ and RM drafted the manuscript, which was reviewed and approved by all authors.

## CONFLICT OF INTEREST STATEMENT

None declared.

## Supporting information


Data S1.

Data S2.

Data S3.

## Data Availability

The data supporting the findings of this study, specifically the N‐glycoproteomics data, are included in the supplemental tables.
