# Supplementary material for: Elevated N‐glycosylated cathepsin L impairs oocyte function and contributes to oocyte senescence during reproductive aging
Source: Aging Cell. 2024 Nov 4;24(2):e14397. doi: 10.1111/acel.14397 (PMC11822660; doi:10.1111/acel.14397)
Supplement: Supplementary file 1 — Data S1. [file ACEL-24-e14397-s003.docx]

**Supple Figure legends**


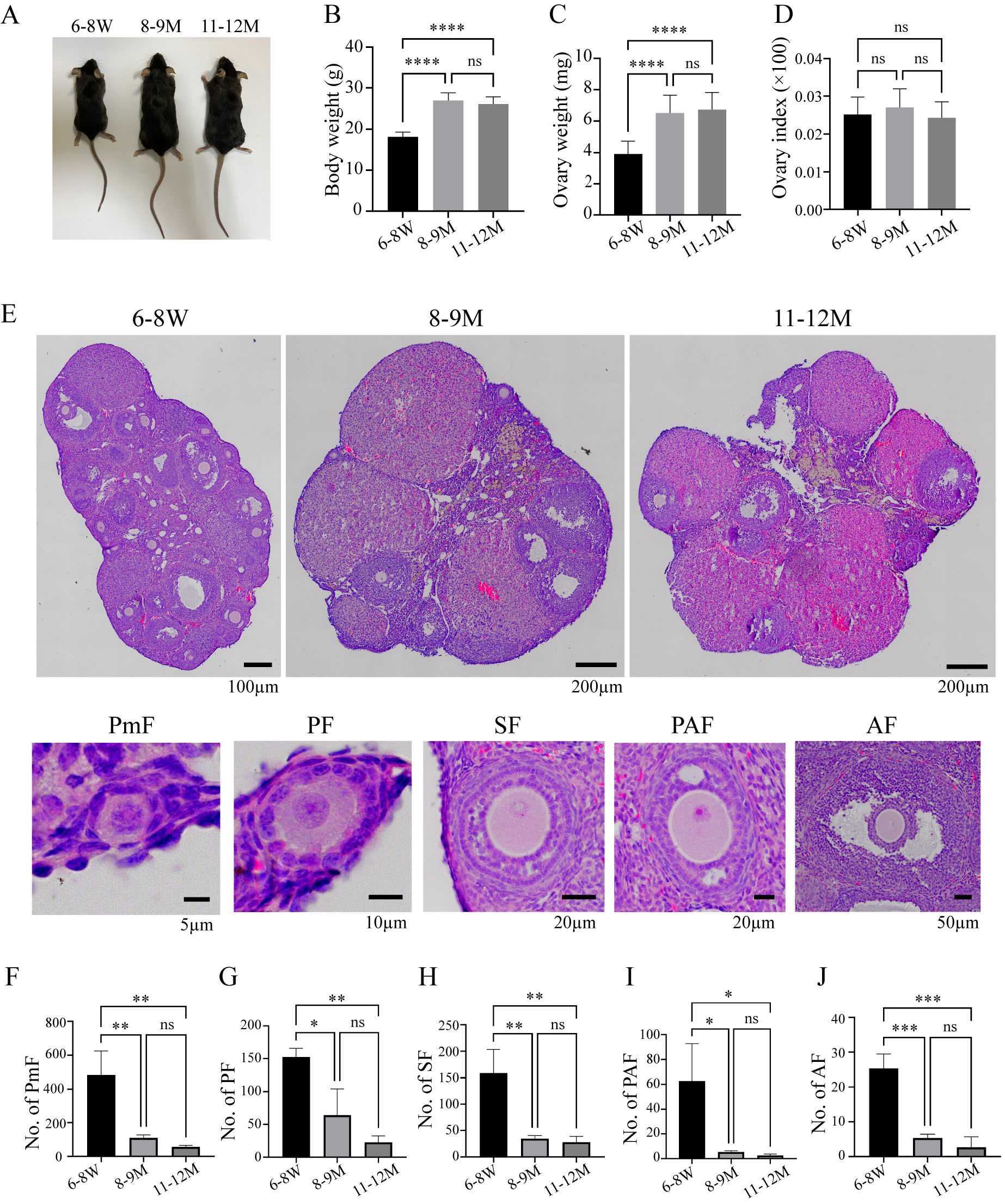


**Figure S1 Evaluation of ovarian function in 6–8 W, 8–9 M, and 11–12 M mice (Related to Figure 1).**

1. Representative images of 6–8 W, 8–9 M and 11–12 M mice.
2. Mouse body weights across the three age groups.
3. Ovary weights across the three age groups.
4. Ovary indexes across the three age groups.
5. H&E staining of ovarian tissue sections across the three age groups.

(F–J) Follicle count and comparison at different developmental stages.

Data in panels (B)-(D), and (F)-(J) are presented as mean ± SEM.

PmF, primordial follicle; PF, primary follicle; SF, secondary follicle; PAF, preantral follicle; AF, antral follicle; ns, not significant; **p <* 0.05; ** *p <* 0.01; *** *p <* 0.001; **** *p <* 0.0001.

**
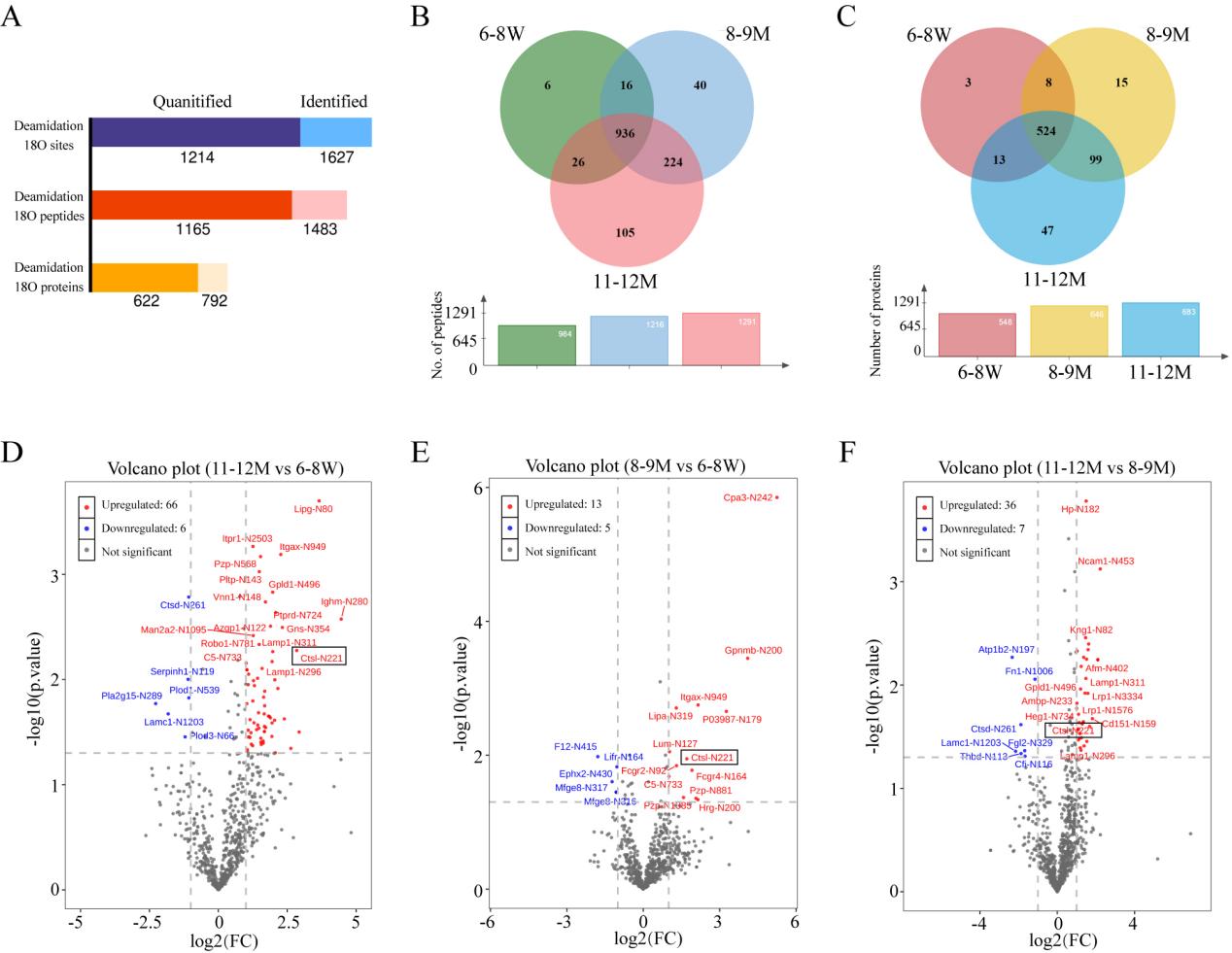
**

**Figure S2** **N-glycoproteomics analysis in ovaries of mice during the aging process (Related to Figures 1–2).**

1. Identification of the number of N-glycosylated sites, N-glycopeptides, and N-glycoproteins.
2. Venn diagram showing the intersection of N-glycopeptides in ovaries of mice aged 6–8 W, 8–9 M and 11–12 M.
3. Venn diagram showing the intersection of N-glycoproteins across the three age groups.
4. Volcano plot displaying DE N-glycopeptides in ovaries between 6–8 W and 11–12 M mice (red, upregulated; blue, downregulated). Some highly DE N-glycopeptides were listed.
5. Volcano plot displaying DE N-glycopeptides in ovaries between 6–8 W and 8–9 M mice (red, upregulated; blue, downregulated). Some highly DE N-glycopeptides are listed.
6. Volcano plot displaying DE N-glycopeptides in ovaries between 8–9 M and 11–12 M mice (red, upregulated; blue, downregulated). Some highly DE N-glycopeptides are listed.


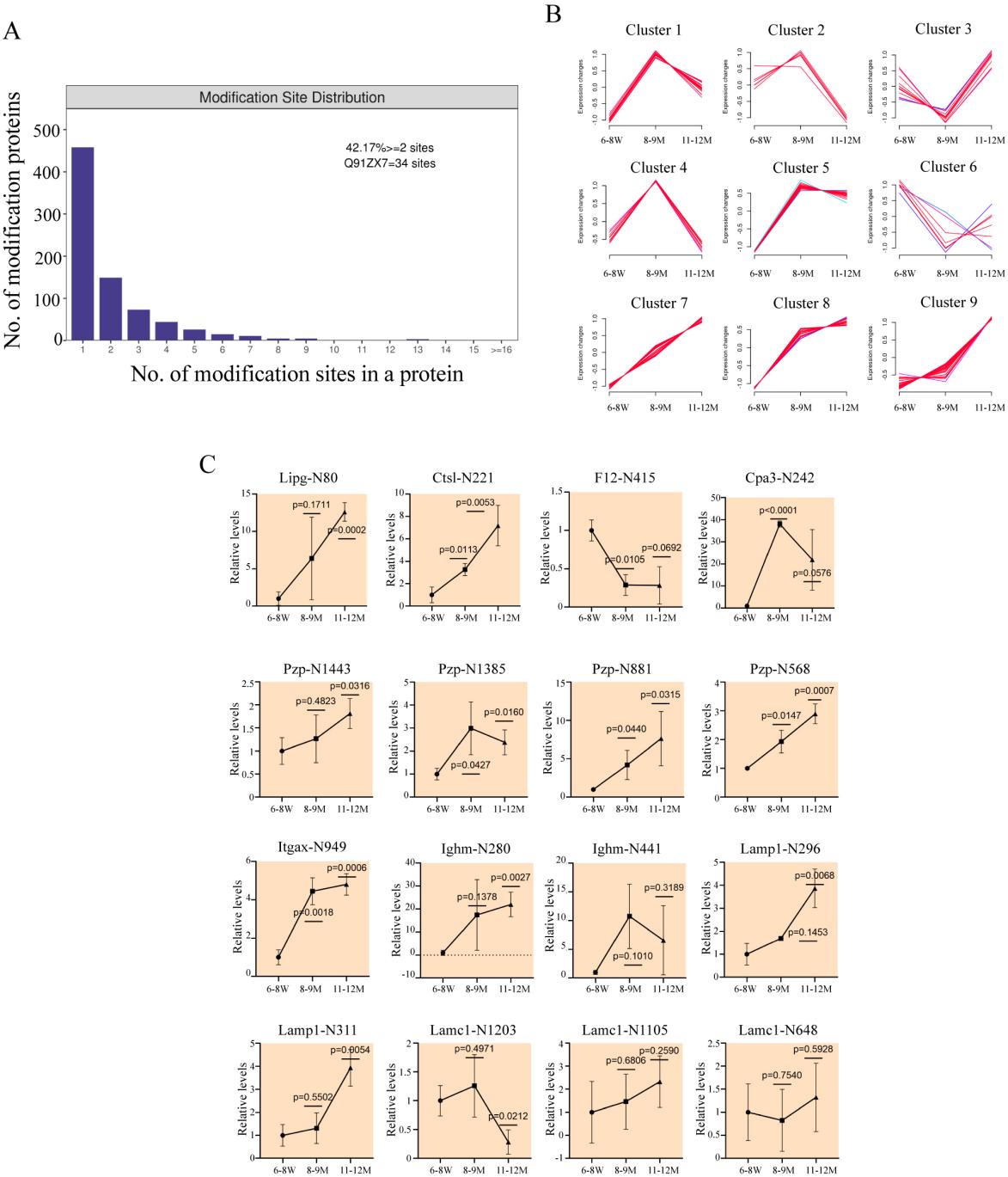


**Figure S3 N-glycoproteomic data in ovaries of mice during the aging process** **(Related to Figures 1-2).**

1. Histogram displaying the distribution of N-glycosylated sites per protein across samples.
2. Clustering analysis of DE N-glycopeptides using Mfuzz across the three age groups.
3. Relative levels of representative N-glycopeptides in mouse ovaries from three developmental stages (6–8 W, 8–9 M, and 11–12 M).


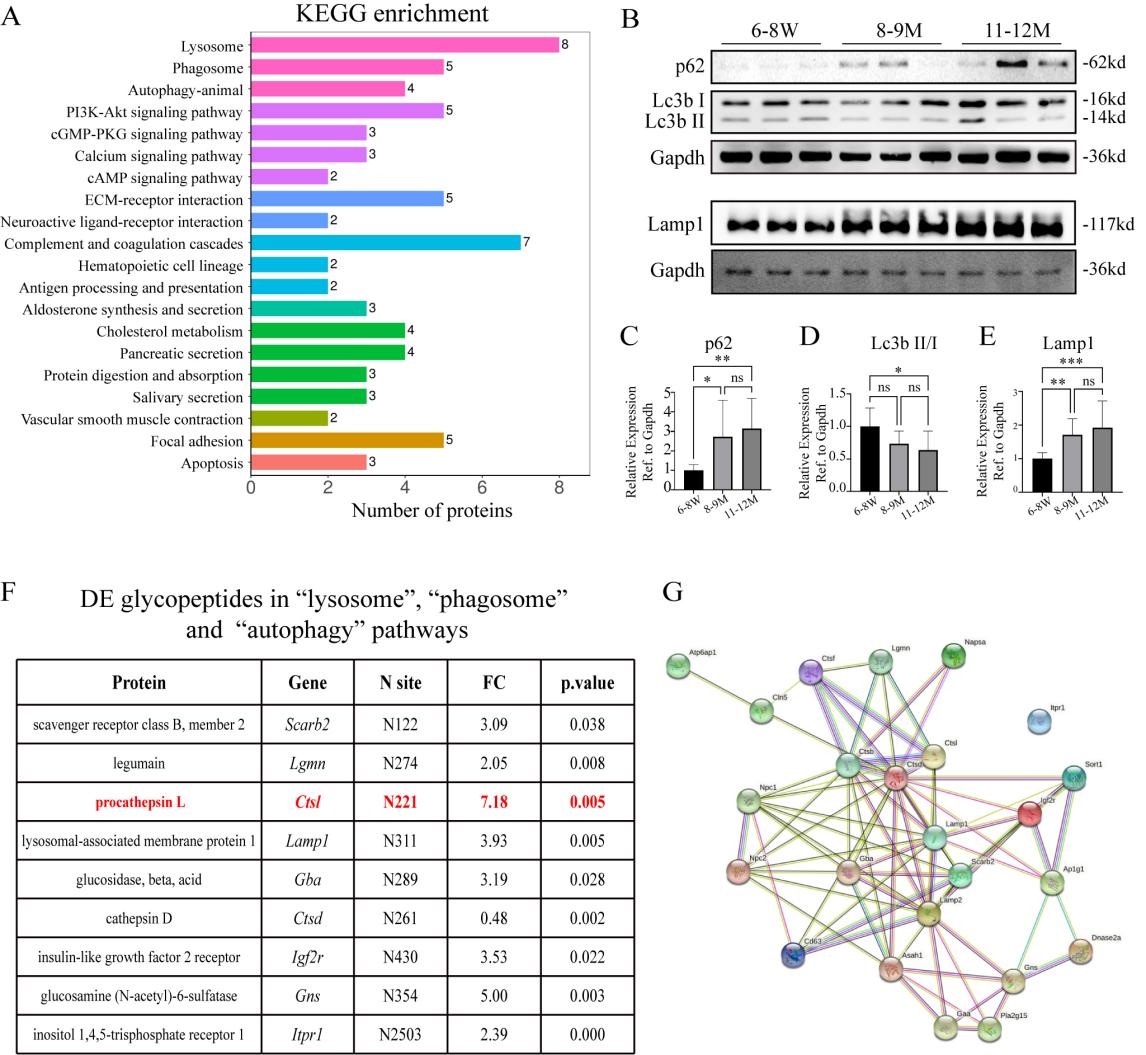


**Figure S4 Enrichment analysis and IB verification of N-glycoproteomic data in aging mouse ovaries** **(Related to Figures 1-2).**

1. KEGG terms for the enrichment of DE N-glycoproteins across the three age groups (6–8 W, 8–9 M, and 11–12 M).
2. IB analysis validating the N-glycoproteomic results through the demonstrated levels of p62, Lc3b, and Lamp1.

(C–E) Protein level quantifications of p62 (C), Lc3b II/I (D), and Lamp1(E).

1. List of DE N-glycopeptides associated with the "lysosome," "phagosome," and "autophagy" pathways.
2. PPI network of DE N-glycoprotein associated with the "lysosome," "phagosome," and "autophagy" pathways.

IB, immunoblotting; PPI, protein-protein interaction; ns, not significant; **p <* 0.05; ** *p <* 0.01; *** *p <* 0.001.


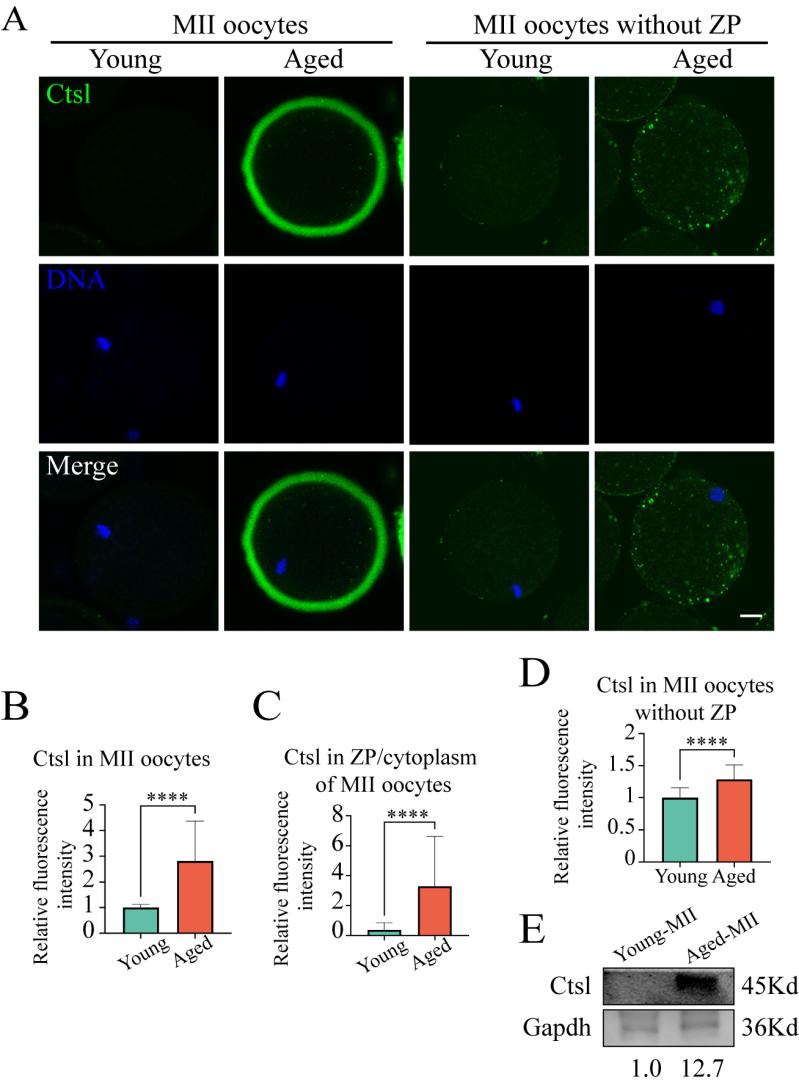


**Figure S5 Ctsl expression profiles in MII oocytes (Related to Figures 2).**

1. Representative images of Ctsl in young and aged MII oocytes with or without ZP. Scale bar, 10 µm. Green, Ctsl; blue, DNA.
2. FI of Ctsl signals between young (n=31) and aged (n=27) MII oocytes.
3. ZP/Cytoplasm fluorescence ratio of Ctsl in young (n=31) and aged (n=27) MII oocytes.
4. FI of Ctsl signals without ZP in young (n=27) and aged (n=23) MII oocytes.
5. IB analysis of Ctsl levels in young (n=80) and aged (n=80) MII oocytes.

Data in panels (B)-(D) are presented as mean±SEM.

MII, metaphase II; ZP, zona pellucida; FI, fluorescence intensity; **** *p <* 0.0001.


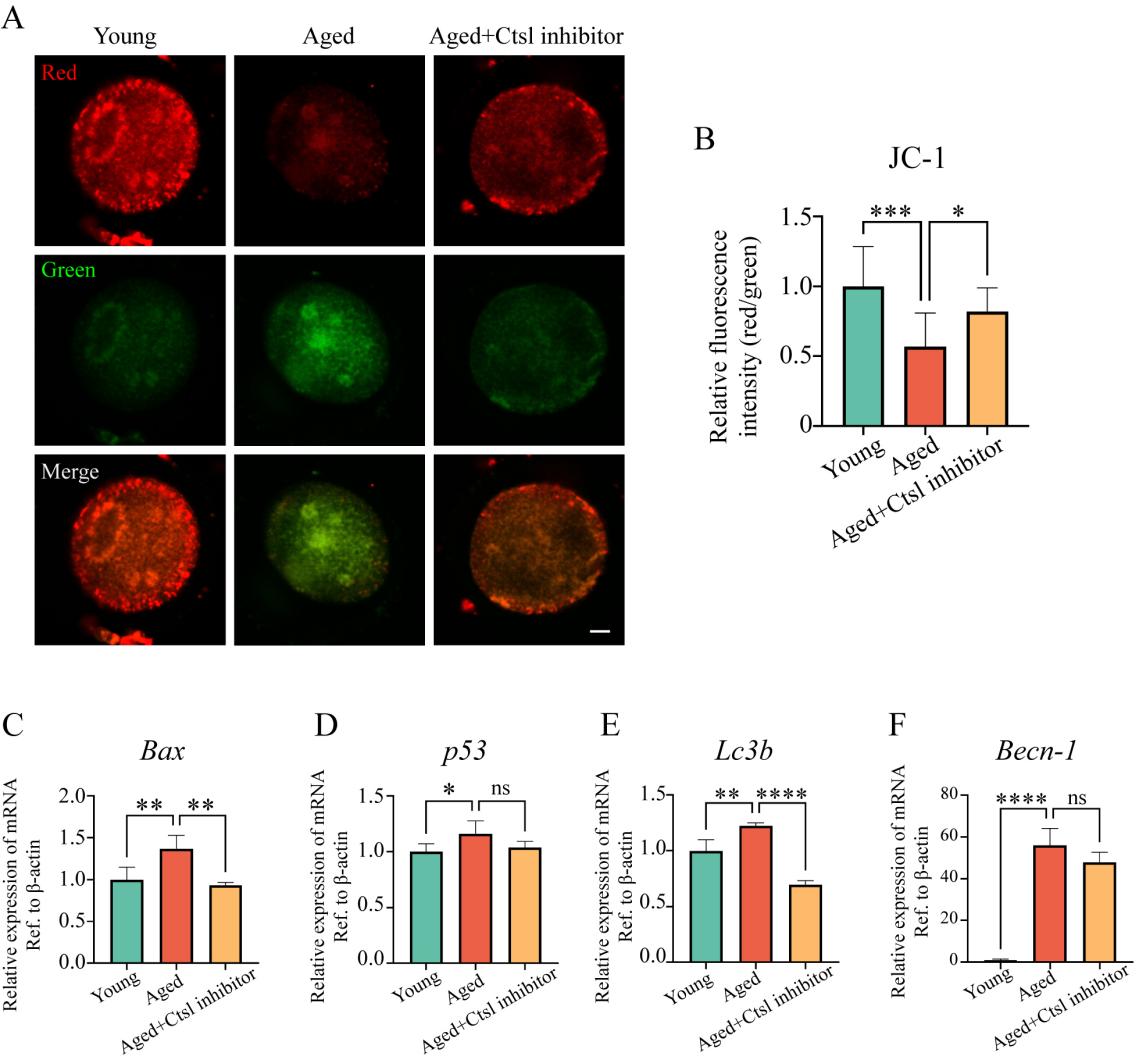


**Figure S6 Effect of Ctsl inhibitor supplementation on mitochondrial function, apoptosis and autophagy in aged mice (Related to Figure 4).**

1. Measurement of MMP (ΔΨm) using JC-1 staining in the three experimental groups (red, high ΔΨm; green, low ΔΨm). Scale bar, 10 µm.
2. The ratio of red to green FI was calculated in the three experimental groups: young (n=30), aged (n=22), and aged+Ctsl inhibitor (n=22).
3. Verification of *Bax* expression in young, aged, and aged + Ctsl inhibitor-treated oocytes using RT-qPCR.
4. Verification of *p53* expression in the three experimental groups using RT-qPCR.
5. Verification of *Lc3b* expression in the three experimental groups using RT-qPCR.
6. Verification of *Becn-1* expression in the three experimental groups using RT-qPCR.

MMP, mitochondrial membrane potential; ns, not significant; **p <* 0.05; ** *p <* 0.01; *** *p <* 0.001; **** *p <* 0.0001.


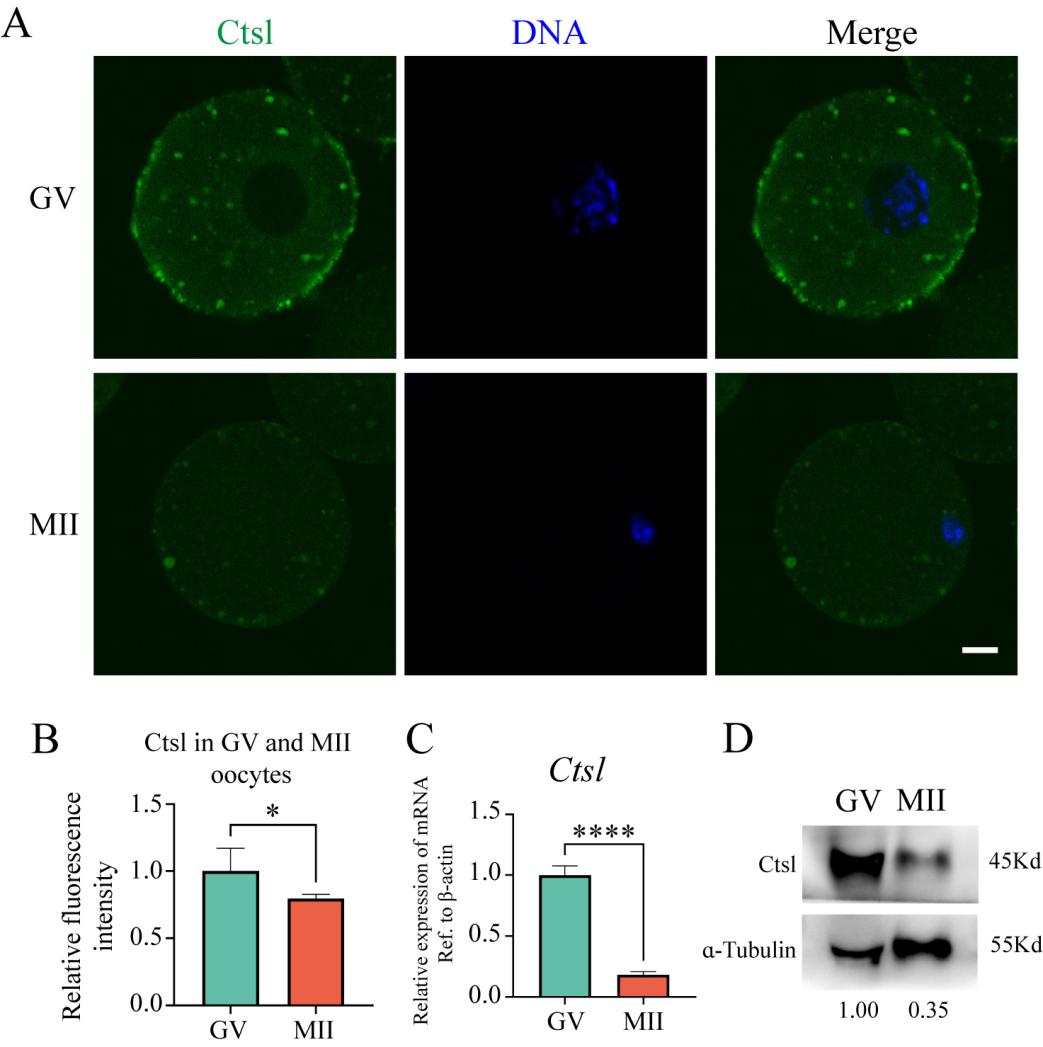


**Figure S7 Analysis of Ctsl expression in GV and MII oocytes (Related to Figure 5).**

1. Imaging of Ctsl expression in GV and MII oocytes. Scale bar, 10 µm.
2. FI of Ctsl signals between GV and MII oocytes.
3. Expression of *Ctsl* in GV and MII oocytes using RT-qPCR.
4. IB analysis displaying the expression of Ctsl in GV and MII oocytes.

**p <* 0.05,*****p <* 0.0001.
