# Supplementary material for: Elevated N‐glycosylated cathepsin L impairs oocyte function and contributes to oocyte senescence during reproductive aging
Source: Aging Cell. 2024 Nov 4;24(2):e14397. doi: 10.1111/acel.14397 (PMC11822660; doi:10.1111/acel.14397)
Supplement: Supplementary file 2 — Data S2. [file ACEL-24-e14397-s002.docx]

**Table S1 Primer sequences of genes for RT-qPCR. Related to Figure S5.**

| **Gene** | **Primer sequence** |
| --- | --- |
| *Bax* | F:AGACAGGGGCCTTTTTGCTAC |
|  | R:AATTCGCCGGAGACACTCG |
| *p53* | F:CCCCTGTCATCTTTTGTCCCT |
|  | R:AGCTGGCAGAATAGCTTATTGAG |
| *Lc3b* | F:TTATAGAGCGATACAAGGGGGAG |
|  | R:CGCCGTCTGATTATCTTGATGAG |
| *Becn-1* | F:ATGGAGGGGTCTAAGGCGTC |
|  | R:TGGGCTGTGGTAAGTAATGGA |
| *β-actin* | F:AGCCATGTACGTAGCCATCC |
|  | R:CTCTCAGCTGTGGTGGTGAA |

**Table S2 Small interfering RNA sequences. Related to Figure 5.**

| **Gene** | **siRNA Sequence** |
| --- | --- |
| *si-Ctsl_001* | ACATGACCAATGAGGAATT |
| *si-Ctsl_002* | CTACTATGAACCCAACTGT |
| *si-Ctsl_003* | GGCTATGAAGGAACAGATT |
