# Supplementary material for: Elevated N‐glycosylated cathepsin L impairs oocyte function and contributes to oocyte senescence during reproductive aging
Source: Aging Cell. 2024 Nov 4;24(2):e14397. doi: 10.1111/acel.14397 (PMC11822660; doi:10.1111/acel.14397)
Supplement: Supplementary file 3 — Data S3. [file ACEL-24-e14397-s001.docx]

**Supplementary materials and methods**

1. **Histological examination and follicle counting**

Ovarian tissues were harvested from the mice, immobilized overnight in 4% paraformaldehyde at 4℃, and subsequently embedded in paraffin. The tissues were sectioned at 5 µm for staining with hematoxylin and eosin (H&E), a process guided by established protocols from previous research (S. Wang et al., 2020).

In the immunohistochemical analysis, ovarian sections underwent an overnight incubation with mouse anti-Ctsl monoclonal antibody (mAb) (1:100; sc-390367, Santa Cruz Biotechnology, USA) at 4℃. This process was followed by a 1-h incubation with a goat anti-mouse IgG (H+L) secondary antibody conjugated to horseradish peroxidase (HRP) (1:500; 31430, Thermo Fisher Scientific, USA) at ambient temperature.

1. **Serum hormone test**

Blood samples from female mice of different age were collected by enucleating of eyeball and allowed to rest at room temperature for 2 h before being centrifuged at 1800 g for 15 min. The separated sera were preserved at -80℃ until required for analysis. Quantitative measurement of serum hormones, including AMH, FSH, LH, E2, Prog, and Ctsl, was performed using ELISA kits (Shanghai Enzyme-linked Biotechnology Co., Ltd) in accordance with established protocols.

1. **Ovarian Tissue Collection and glycoproteomics sample preparation**

Ovaries from female mice at developmental stages of 6–8 W, 8–9 M, and 11–12 M were harvested and immediately processed. Tissue samples were homogenized in sodium dodecyl sulfate, dithiothreitol (SDT) buffer containing 4% sodium dodecyl sulfate (SDS), 100 mM dithiothreitol (DTT), and 150 mM Tris-HCl at pH 8.0, followed by sonication and boiling for 15 min. The lysates were then centrifuged at 14000 g for 40 min, and the supernatant was retrieved for protein quantification using the BCA Protein Assay Kit (Bio-Rad, USA).

For glycoproteomic analysis, 200 µg of proteins from each sample were processed by mixing with 30 µl of SDT buffer and subjected to extensive ultrafiltration in UA buffer (8 M urea, 150 mM Tris-HCl, pH 8.0) to eliminate detergent, DTT, and low-molecular-weight impurities. The samples were then alkylated by adding 100 µl of 100 mM iodoacetamide in UA buffer and incubated without light exposure for 30 min. Proteins were enzymatically digested into peptides using 4 µg of trypsin in 40 µl of 25 mM NH_4_HCO_3_ buffer at 37℃ overnight. The digested peptides as filtrates were subsequently captured on C18 Cartridges (Empore™ SPE Cartridges C18; standard density; bed I.D. 7 mm, volume 3 ml, Sigma) for desalting, concentrated *via* vacuum centrifugation, and re-suspended in 40 µl of 0.1% formic acid (FA).

1. **N-glycopeptide enrichment and deglycosylation in heavy water (H_2_^18^O)**

The enrichment of N-glycopeptides was carried out utilizing a combination of lectins containing concanavalin A (ConA), wheat germ agglutinin (WGA), and ricinus communis agglutinin (RCA) as described (Zielinska, Gnad, Schropp, Wisniewski, & Mann, 2012; Zielinska, Gnad, Wisniewski, & Mann, 2010). The peptides were mixed with these lectins at a weight-to-weight ratio of 1:2 (lectin to protein). This preparation was then placed into YM-30 filter units. Following a 1-h incubation at ambient temperature, peptides not bound to the lectins were separated by centrifugation at 14,000 g for 10 min. To minimize false positives due to deamidation, the bound peptides were thoroughly washed with binding buffer and NH_4_HCO_3_ buffer in H_2_^18^O three times prior to enzymatic treatment. After washing, peptide-N-glycosidase F (PNGaseF) was introduced by transferring the filter units to a new tube, adding 40 µl of 25 mM NH_4_HCO_3_ in H_2_^18^O containing 3 µg PNGaseF, and incubating at 37℃ for 3 h. Finally, deglycosylated peptides were eluted *via* centrifugation at 14,000g for 10 min.

1. **Liquid chromatography (LC)-MS/MS protocols**

The peptides were analyzed using a Q Exactive HF/HFX mass spectrometer (Thermo Scientific) connected to an Easy-nLC system (Proxeon Biosystems, Thermo Fisher Scientific) over a period of 120 min. Initial peptide loading was onto a reverse-phase trap column (Thermo Scientific Acclaim PepMap100, 100 µm × 2 cm, nanoViper C18), followed by elution to a C18-reversed phase analytical column (Thermo Scientific Easy Column, 10 cm long, 75 µm inner diameter, 3 µm resin) using buffer A (0.1% FA). The eluted peptides were separated through a linear gradient of buffer B (84% acetonitrile, 0.1% FA), maintained at a 300 nl/min flow rate *via* IntelliFlow technology. MS analysis was conducted in positive ion mode using a data-dependent top10 acquisition strategy that dynamically selected the most abundant precursor ions from a survey scan (300-1800 m/z) for subsequent higher-energy collisional dissociation (HCD) fragmentation. The setup included an automatic gain control (AGC) target of 3e6, a maximum injection time of 10 ms, and a dynamic exclusion period of 40.0 s. The mass resolution for survey scans was established at 70,000 at m/z 200, with HCD spectra resolution at 17,500 at m/z 200 and an isolation width of 2 m/z. The normalized collision energy was set at 30 eV, and an underfill ratio of 0.1% was defined to optimize ion filling. The peptide recognition mode of instrument was activated to enhance peptide identification and quantification.

1. **Identification of N-glycoproteins and bioinformatic analysis**

The raw data generated by MS for each sample underwent computational analysis using MaxQuant software (version 1.6.14.0), which facilitated the search against the UniProt mouse proteome database (17097 sequences updated as of 2022/01/04). The analysis parameters were defined: a precursor mass tolerance of 20 ppm; trypsin as the digestive enzyme allowing up to two missed cleavages; and variable modifications including methionine oxidation (+15.9949 Da) and asparagine deamidation in H_2_^18^O (^18^O tag of Asn, +2.9890 Da). A false discovery rate (FDR) of 1% was applied to both peptides and proteins to ensure high accuracy. Statistical significance in protein abundance variations among different groups was assessed using one-way analysis of variance (ANOVA), where a p-value of less than 0.05 and a fold change exceeding 2.0 were criteria for significance.

Hierarchical clustering was executed employing Cluster 3.0, which can be accessed at http://bonsai.hgc.jp/~mdehoon/software/cluster/software.htm, along with Java Treeview software, available at http://jtreeview.sourceforge.net. The Euclidean distance algorithm was employed for similarity measurement, and the average linkage clustering algorithm, which utilizes the centroids of observations, was selected for clustering operations. Normalization of DE glycopeptides was achieved by z-score transformation to standardize the data for further analysis. GO and KEGG pathway analyses were facilitated by the DAVID bioinformatics resources (Huang da, Sherman, & Lempicki, 2009). Enrichment analysis was conducted using Fisher's exact test with the complete dataset of quantified proteins serving as the background dataset. Adjustments for multiple comparisons were made using the Benjamini-Hochberg procedure, with significance thresholds set at p-values below 0.05.

Furthermore, the study extended into the PPI network, where data regarding the proteins of interest were extracted from the IntAct database (http://www.ebi.ac.uk/intact/) *via* STRING software (http://string-db.org/). The results were downloaded in XGMML format and imported into Cytoscape software (http://www.cytoscape.org/, version 3.2.1) for visualization and further analysis of functional PPI networks.

1. **IF and confocal imaging**

The oocytes were immobilized in paraformaldehyde for 30 min and permeabilized using 0.5% Triton X-100 for 20 min at ambient temperature. Subsequent to three washes in PBS containing 0.1% Tween-20 and 0.01% Triton X-100, the oocytes were blocked in PBS containing 1 mg/ml BSA for 1 h to prevent non-specific binding. The oocytes were incubated overnight at 4℃ with a cocktail of primary antibodies: mouse anti-Ctsl mAb (1:100; sc-390367, Santa Cruz Biotechnology), mouse anti-α-tubulin-FITC mAb (1:250; F2168, Sigma-Aldrich), and rabbit anti-LAMP1 mAb (1:100; ab208943, Abcam, Cambridge, UK). Following addidtional three washes, the oocytes underwent incubation with secondary antibodies (1:100; ZSGB-Bio, China): FITC-conjugated goat anti-mouse IgG (H+L) (ZF0312) or Rhodamine-conjugated goat anti-rabbit IgG (H+L) (ZF0316) for 1 h at ambient temperature. For nuclear visualization, oocytes were stained with Hoechst 33342 (C1026, Beyotime, Hangzhou, China) at ambient temperature for 15 min. Following a final wash to remove excess stain, the oocytes were mounted on slides and visualized using a Zeiss LSM 810 confocal microscope (Germany).

Active mitochondrial function in the oocytes was visualized by incubating them in M2 medium containing 200 nM MitoTracker Red CMXRos (M7512, Thermo Fisher Scientific) at 37℃ in a controlled 5% CO_2_ environment for 30 min. Subsequently, the oocytes were rinsed thrice and relocated to a DPBS solution enriched with BSA (1 mg/ml) for confocal microscopy examination.

MMP was quantitatively assessed using a JC-1 (C2003S, Beyotime) based MMP assay following the manufacturer's instructions. The oocytes were immersed in the prepared JC-1 working solution (2 μl 500× JC-1, 900 μl ddH_2_O, 100 μl 10× incubation buffer) at 37℃ for 20 min. The oocytes were cleansed in 1× incubation buffer and immediately analyzed under a confocal microscope. The shift of JC-1 dye from green (529 nm) to red (590 nm) fluorescence under varying mitochondrial potentials facilitated a quantitative evaluation of MMP through the intensity ratio of red to green fluorescence.

ROS accumulation in oocytes was quantified using an ROS Assay Kit (E004-1-1, Jiancheng, Nanjing, China) following the manufacturer's protocol. The oocytes underwent a 30-min incubation in a dichlorofluorescein diacetate (DCFHDA; 10 μM)-enriched DPBS solution under a 5% CO_2_ atmosphere at 37°C. After three washes, the oocytes were visualized using a confocal microscope.

Apoptotic activity in oocytes was detected using the Annexin-V Staining Kit (C1062M, Beyotime). Initially, the oocytes were exposed to Tyrode's solution (T1788, Sigma-Aldrich) to eliminate the ZP. Following a brief washing period, the oocytes were stained in a mixture of 90 µl binding buffer and 10 µl Annexin-V-FITC, and incubated without light exposure for 30 min. The stained oocytes were then visualized under a confocal microscope to assess apoptotic markers.

Consistent IF protocols and confocal microscopy settings were followed for accurate comparison of fluorescence signals among various groups. A region of interest (ROI) was designated for each sample, and FI measurements in these ROIs were systematically performed using ImageJ software (NIH, Bethesda, MD, USA).

1. **IB assay**

Ovarian tissues were processed for protein extraction using a standard lysis buffer, followed by disruption through sonication. Protein concentrations were determined with the BCA Protein Assay Kit. Prepared samples were mixed with protein loading buffer and subjected to denaturation at 95°C for 10 min. Similarly, oocytes were disrupted in 4× LDS sample buffer supplemented with protease inhibitors, and the mixture was boiled for 10 min. Proteins were then separated by SDS-PAGE and transferred onto membranes for immunoblot analysis. The membranes were incubated with primary antibody at at 4°C overnight. The primary antibodies employed for detection included: mouse anti-Ctsl mAb (1:250; sc-390367, Santa Cruz Biotechnology), rabbit anti-p62/Sqstm1 mAb (1:10000; ab109012, Abcam), rabbit anti-Lc3b mAb (1:2000; ab192890, Abcam), rabbit anti-Lamp1 mAb (1:1000; ab208943, Abcam), and mouse anti-Gapdh mAb (1:20000; 60004-1-Ig, Proteintech, China). After washing three times with Tris-buffered saline containing Tween 20 (TBST), the blots were probed with a goat anti-mouse IgG-HRP secondary antibody (1:5000; 31430, Thermo Fisher Scientific, USA) or goat anti-rabbit IgG-HRP secondary antibody (1:5000; 31460, Thermo Fisher Scientific, USA) for one hour at ambient temperature.

1. **RNA isolation and quantitative real-time PCR**

Total RNA from MII oocytes, which were matured *in vitro* (20 oocytes per sample), was extracted using the PicoPure^TM^ RNA Isolation Kit (KIT0204, Thermo Fisher Scientific). Subsequent cDNA synthesis was performed using HiScript II Q RT SuperMix for qPCR (+gDNA wiper) (R223-01, Vazyme, Nanjing, China) as prescribed by the product guidelines. For the quantitative assessment of gene expression, real-time PCR was executed using ChamQ SYBR qPCR Master Mix (Q321, Vazyme) on the Cobas Z 480 Real-time PCR system (Roche). Normalization of the expression data was carried out against the *β-actin* gene, and expression differences were quantified using the ΔCq method. Detailed primer sequences are listed in **Table S1**.
